# Precision and geographical prevalence mapping of schistosomiasis and soil-transmitted helminthiasis among school-aged children in selected districts of north-western Tanzania

**DOI:** 10.1186/s13071-022-05547-6

**Published:** 2022-12-29

**Authors:** Humphrey D. Mazigo, Maria M. Zinga, Stella Kepha, Elodie Yard, Kevin McRee-Mckee, George Kabona, Deogratias D. Ngoma, Andreas Nshala

**Affiliations:** 1 School of Medicine, Department of Medical Parasitology, Catholic University of Health and Allied Sciences, 1464, Mwanza, Tanzania; 2 Kenya Medical Research Institute (KEMRI), Nairobi, Kenya; 3 Oriole Global Health, Halifax, Canada; 4 Oriole Global Health Kenya, Nairobi, Kenya; 5 National Neglected Tropical Diseases Control Programme, Ministry of Health, 743, Dodoma, Tanzania; 6 Helen Keller International, 34424, Dar es Salaam, Tanzania; 7 Arise Programme, Crown Agents, London, UK

**Keywords:** Precision mapping, Schistosomiasis, Soil-transmitted helminths, Sub-districts, North-western Tanzania

## Abstract

**Background:**

The identification and mapping of at-risk populations at a lower administrative level than the district are prerequisites for the planning, resource allocation and design of impactful control intervention measures. Thus, the objective of the current study was to conduct sub-district precision mapping of soil-transmitted helminthiasis (STH) and schistosomiasis in 29 districts of north-western Tanzania using the current recommended World Health Organization criteria.

**Methods:**

A cross-sectional survey was conducted in 145 schools between March and May 2021. A urine filtration technique was used for the quantification of *Schistosoma haematobium* eggs, whereas quantification of *Schistosoma mansoni* and STH eggs was done using the Kato–Katz technique. Microhaematuria was examined using a urine dipstick.

**Results:**

The overall prevalences of any STH and schistosome infections were 9.3% [95% confidence interval (95%CI) 8.6–9.9] and 14.6% (95%CI 13.9–15.4), respectively. The overall prevalence of *S. mansoni* was 8.7% (95%CI 8.1–9.3), and 36.4%, 41.6%, and 21.9% of the children had low, moderate, and heavy infections, respectively. The overall prevalence of *S. haematobium* was 6.1% (95%CI 5.5–6.5), and 71.7% and 28.3% of the infected children had light and heavy intensity infections, respectively. The prevalence of microhaematuria was 7.3% (95%CI 6.7–7.8), with males having the highest prevalence (8.4%, *P* < 0.001). The prevalences of *Trichuris trichiura*, *Ascaris lumbricoides* and hookworm were, respectively, 1.3% (95%CI 0.1–1.5), 2.9% (95%CI 2.5–3.3) and 6.2% (95%CI 5.7–6.7). Most of the children infected with STH had light to moderate intensities of infection. The overall prevalence of co-infection with STH and schistosomiasis was 19.1%. The prevalence of schistosomiasis (*P* < 00.1) and STH (*P* < 0.001) varied significantly between schools and sub-districts. *Schistosoma mansoni* and *S. haematobium* were observed in 60 and 71 schools, respectively, whereas any STH was observed in 49 schools. In schools where schistosomiasis was observed, prevalence was < 10% in 90.8% of them, and ranged from ≥ 10% to < 50% in the other 9.2%. In schools where any STH was observed, the prevalence was < 10% in 87.7% of them.

**Conclusions:**

The data reported here show that schistosomiasis and STH are widely distributed around Lake Victoria. In most of the schools where schistosomiasis and STH occurred the transmission thresholds were low. These data are important and need to be taken into consideration when decisions are made on the implementation of the next round of mass chemotherapies for schistosomiasis and STH in Tanzania.

**Graphical Abstract:**

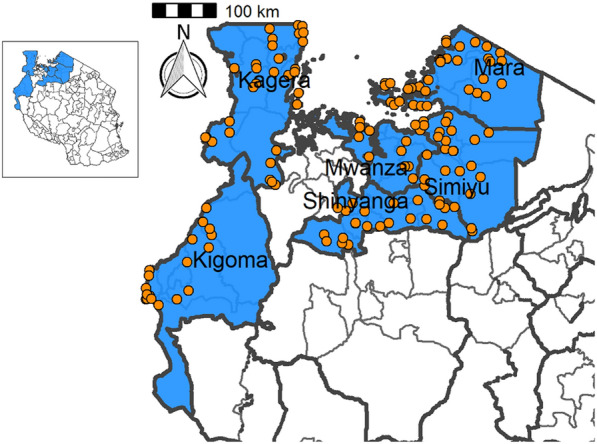

**Supplementary Information:**

The online version contains supplementary material available at 10.1186/s13071-022-05547-6.

## Background

In Tanzania, both urogenital and intestinal schistosomiasis, which are caused by *Schistosoma haematobium* and *Schistosoma mansoni*, respectively, are endemic at varying transmission levels in all administrative regions and districts [1]. The north-western regions surrounding Lake Victoria are endemic for *S. mansoni*, while the areas beyond the lake basin in the same regions are endemic for *S. haematobium. Schistosoma haematobium* is widely distributed at the far end of the southern shoreline of Lake Victoria, while endemic areas for *S. mansoni* are mostly focal [1]. In 2012 in Tanzania, 23.2 million people of the country’s estimated 43.5 million population were estimated to have schistosomiasis, which gave a national prevalence of schistosomiasis of 51.5% [2]. In the same regions, soil-transmitted helminthiasis (STH; *Ascaris lumbricoides*, *Trichuris trichiura* and hookworm) are also endemic and co-occur with schistosomiasis [3]. In endemic areas, school-aged children (SAC) and pre-SAC have the highest prevalences and intensities of STH infections [4]. In the last period of mapping, which was conducted in 2004/2005, the prevalence of STH ranged from 0 to 40% [5].

Mass drug administration (MDA) using praziquantel (PZQ) and albendazole (ALB) is the key strategy for controlling schistosomiasis and STH infections in Tanzania and is used to target SAC within the school environment [6]. During MDA programmes, pre-school children receive only ALB against STH [7]. Parasitological data are used to determine the eligibility of a district(s) for MDA based on the endemicity level [8, 9]. Traditional methods of determining treatment strategies recommended by the World Health Organization (WHO) involve collecting data at the district level (i.e. the implementation unit). Parasitological data are obtained by screening 50 SAC per school from a sub-sample of up to five schools selected per district [8, 9]. The mean prevalence of schistosomiasis allows a district to be classified as follows: non-endemic (0%), low (< 10%), moderate (10% to < 50%), or high (> 50%) risk [8, 9]. Similarly, the mean prevalence of STH is used to categorize districts as low risk areas (prevalence ≤ 20%) and high risk areas (prevalence ≥ 50%) [10]. However, this approach can result in the misclassification of districts due to the focal nature of schistosomiasis, and lead to either over-treatment or under-treatment if the decision to implement MDA is based on the generated evidence [9]. This conventional mapping approach is currently not recommended for achieving schistosomiasis elimination goals and endemic country targets. Recognizing this, the WHO have recommended a new mapping approach, termed precision mapping/granular mapping [11]. Precision mapping is defined as sampling at a much finer geographical resolution, potentially examining all schools within every subunit in each implementation unit in order to eliminate errors caused by missing the focal variation in schistosomiasis [11]. It is worthwhile noting that the current mapping approach is recommended for mapping schistosomiasis only. However, because schistosomiasis and STH are co-endemic in Tanzania and the same diagnostic technique is used for them [the Kato–Katz (KK) technique], STH infections were included in this study. While the conventional mapping approach considers a district as an implementation unit [9], the current approach considers sub-district(s)/ward(s) as an implementation unit [11]. This approach allows the mapping of more geographical points to deepen our understanding of the distribution of schistosomiasis and STH at district level and helps in guiding the decision-making process [11]. It should be noted that, due to financial constraints, mapping all sub-districts/wards/clusters independently in a single district may not be possible. Thus, integrating existing ecological knowledge on factors which influence/favour transmission of schistosomiasis in a particular area or across different district sub-units/wards/clusters may help in reducing mapping costs by allowing for the combination of several sub-districts/wards/clusters in similar ecological zones into a mapping unit [11, 12]. Communities or schools located in close proximity to natural water bodies such as lakes and rivers, or man-made water bodies such as dams, irrigation systems, and rice-farming communities, should be highly suspected of having members or pupils with the disease, and previous knowledge of schistosomiasis transmission in them should also be taken into consideration when selecting the areas for mapping [9, 11, 13]. The selection of school(s) or communities should ensure geographical representation of the same or different localities within a health district [9, 11, 12]. Random selection of schools is not recommended [9, 11].

In north-western Tanzania, mapping using parasitological data on primary school children was last conducted in 2004/2005, and showed that the prevalence of schistosomiasis ranged from 12.7% to 87.6% [5]. Despite almost 15 years of MDA in Tanzania, there is limited information on post-MDA geographical prevalence for many of the endemic districts [14]. Therefore the following questions remain unanswered: (i) which areas still have high prevalences of STH and schistosomiasis and may require additional resources for their effective control; and (iii) which sub-districts/wards have sufficiently reduced transmission that they no longer require MDA, or require a revised MDA strategy? Thus, before opting for different MDA strategies for different sub-districts/wards, a better understanding of the current post-MDA geographical prevalence of schistosomiasis and STH is needed [15]. Therefore, the aim of the present study is to determine the geographical prevalence, intensity of infection and geographical distribution of schistosomiasis (both urogenital and intestinal schistosomiasis) and STH among SAC at the sub-district, or ward level, in 29 selected districts of north-western Tanzania.

## Methods

### Study setting

**Fig. 1:**
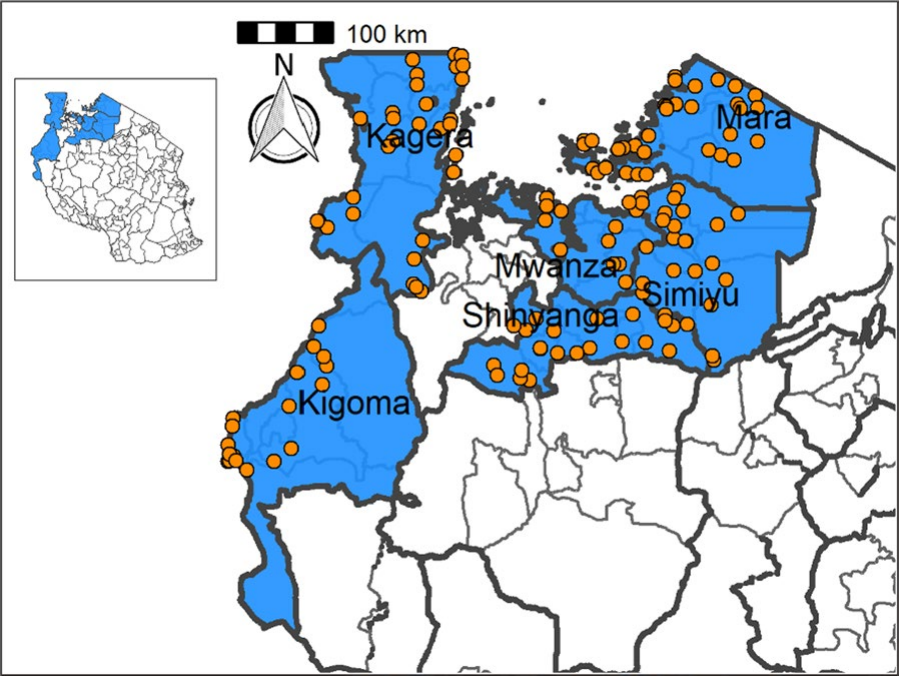
Locations of the schools within the studied districts

North-western Tanzania comprises Mwanza, Mara, Kigoma, Kagera, Simiyu and Shinyanga regions (Fig. 1). Mara is located on the eastern, Simiyu on the south-eastern, Mwanza on the southern and Kagera on the south-western shoreline of Lake Victoria. Shinyanga is located to the south of Lake Victoria, and does not border the lake. Kigoma is located south-west of Lake Victoria and is located on the north-eastern shoreline of Lake Tanganyika (Fig. 1). All of these regions are traditionally endemic for STH, *S. mansoni*, STH and *S. haematobium* [1]. The districts bordering the southern shoreline of Lake Victoria are endemic for *S. mansoni*, whereas mixed infections of *S. haematobium* and *S. mansoni* have been reported for more southerly districts outside the basin, with *S. haematobium* being the predominant species [1]. STH are widely distributed in the region of Lake Victoria, with hookworm being common along the shoreline and in dry areas, while *T. trichiura* and *A. lumbricoides* are typically found in wet areas on the western side of the lake [3, 5]. Pre-MDA schistosomiasis mapping between 2004 and 2005 categorized the selected districts as having low to moderate endemicity, with prevalence ranging from 10 to 49.9% [5]. That survey included 5201 SAC from 86 schools selected from the same districts as those included in the present study, and the overall prevalence of *S. haematobium* was 20.7% (1075/5201) [5]. The prevalences of *S. mansoni*, *A. lumbricoides*, *T. trichiura* and hookworm were 0.8%, 0.28%, 0.13% and 31.8%, respectively [5].

The study reported here was undertaken in 29 districts of Mwanza, Mara, Kagera, Kigoma, Shinyanga and Simiyu regions. The ecology of these regions is characterized as tropical, and the rainfall is bimodal, with the long rainy season starting in February/March and lasting until the end of May and the short rainy season starting in October and lasting until December. The area receives between 1100 mm to 1600 mm of rainfall annually and the average temperature is 25–28 °C. The area is characterized by a flat landscape, an extension of the Serengeti plains, to the south of Lake Victoria, and V-shaped hills and valleys to the west of Lake Victoria. Seasonal and permanent rivers flow down the plains to Lake Victoria and Lake Tanganyika. The regions’ predominant livelihoods are livestock keeping, rice farming, and cotton and maize cultivation. The major tribe is the Wasukuma, a Bantu group, and the other, smaller, tribes in the area are the Waha, Wanyatunzu, Wakurya, Wakerewe, Wajita and Wazinza.

### Study population and inclusion and exclusion criteria

Primary school children aged 9–13 years, who attended the selected schools, were targeted for the study as they comprise the high-risk age group for STH and schistosomiasis in endemic areas [16, 17]. Inclusion criteria for the children were as follows: 9–13 years of age, permanent residents of the selected areas, no history of anthelminthic treatment in the past 6 months, submitted a single urine and stool sample, and provided signed parental consent for participation in the study.

### Sampling and sample size

A ward(s) within a defined ecological zone was used as a sampling unit. An ecological zone was defined as areas located within a similar distance from bodies of water (e.g. rivers, lakes, streams, ponds, dams, irrigation channels) within a locality [9, 12, 13]. An interactive mapping process was used to identify and map all the water bodies in the selected wards. All of the schools in each ward within the selected district were mapped. For the selection of the wards, each district was sequentially subdivided into lower administrative levels such as wards and villages. Then the district was divided into one, two or three different ecological zones based on its proximity to water bodies (near, < 1 km; medium, 1-5 km; and far, > 5 km) [12, 13]. All the wards and schools within a particular ecological zone were identified according to WHO guidelines [9]. From five to 15 schools were sampled from each ecological zone. It is worth noting that the selected schools met the criteria of the possible mapping scenarios (known, unknown and suspected) as described in the WHO guidelines; at least five schools were selected from each category. To ensure representation of each ward/cluster, selected schools in different ecological zones were mutually exclusive, thus a school was only included for one administrative level/ward/cluster. In the case where several wards/clusters were located in the same ecological zone (where the transmission of schistosomiasis was likely to be similar), the wards/clusters were combined into a single mapping unit and less than 15 primary schools were selected from this mapping unit, while ensuring representation of each ward/cluster [9]. After selecting the schools, a simple random sampling technique was used to select 60 SAC (30 boys and 30 girls) between the ages of 9 and 13 years, using the lottery method (drawing of numbers in a random fashion) [13, 18]. The WHO recommends the inclusion of 50 SAC from each school in a study [13]. To take into consideration a 16% rate of non-response/dropout of participants during a study, an additional 10 SAC were included in addition to the number recommended by the WHO.

### Recording of demographic information of participants

Age, sex, village/ward name, school name and school identification number were recorded in the master enrolment list. The barcode of a sticker with the specific number of each participant was used as a unique identification number on the biodata form for each study participant.

### Parasitological examination of stool and urine samples for schistosomiasis

#### Stool and urine sample collection

On the day of sample collection, the signed informed consent forms were collected from all the eligible children, who also gave their assent for inclusion in the study. All the participants were assigned an individual study identification number, which was used as an identifier for the sample collection containers, microscopic slides, register, enrolment forms, written informed consent forms and their assent. Each study participant then received an empty, clean, labelled container and tissue paper and was instructed on how to hygienically collect urine and stool samples. After sample collection, the participants were instructed to wash their hands with soap (liquid medicated soap provided by the study team).

#### Examination of *S. mansoni* eggs using the KK thick technique

A single fresh stool sample was collected from each participating child using a stool container, and duplicate KK thick smears were prepared using a slide template of 41.7 mg per thick smear [19]. The KK smears were examined within 1 h of preparation for the presence of hookworm eggs, and after 24 h, the slides were examined for the presence of eggs of *S. mansoni* and other STH (*T. trichiura*, *A. lumbricoides*) by two independent laboratory technicians. For quality assurance, 20% of all the positive and negative slides were re-examined by a third examiner blinded with respect to the results of the first two examiners.

#### Examination of *S. haematobium* infection using a urine filtration technique

A single urine sample was collected from all study participants between 10.00 a.m. and 2.00 p.m. within the school environment. The urine samples underwent gross examination for the presence of macrohaematuria and a urine dipstick/urinalysis reagent strip (Mission Expert; ACON, USA) was used to test for the presence of microhaematuria. The microhaematuria results were recorded as per the manufacturer’s instructions (negative, trace +, ++ and +++). After urine filtration, light microscopy was used to examine the urine filters stained with Lugol’s iodine or povidone iodine for the presence of *S. haematobium* eggs [20]. Eggs were counted and the number recorded. For the purpose of quality assurance, a third technician re-examined 20% of all the positive and negative urine filters at the field sites.

### Geographical distribution of infections

To determine the geographical distribution of schistosomiasis and STH prevalence, a Geographic Information Systems/Global Positioning System was employed to map their spatial patterns. Maps were generated using R statistical software (version 2022.02.2+461) via the package ggplot2 to plot point prevalence and choropleth maps based on WHO infection categories. Sub-county- and ward-level shapefiles were imported with the support of the Ministry of Health, and site/village geographical coordinates were recorded via Kobo Collect throughout the data collection process.

### Ethical approval and permission to conduct the study

Ethical approval for this study was sought from the National Ethical Committee, National Institute for Medical Research Tanzania (NIMR/HQ/R.8a/Vol. IX/3481). Further permission was sought from the regional and district administrative authorities of the regions involved in the study. Two days before participating in the study, the children received an informed consent form translated into Kiswahili to give to their parent(s)/guardian(s), who were invited to the school on the day of screening to give their consent. An assent form was developed for children aged 9–13 years that the children could read to understand the study procedures and objectives. The purpose of the study, participation and withdrawal rights, and risks and benefits were explained fully to the participants and their parents/guardians.

On the day of sample collection, only the assenting children whose consent forms had been signed by their parents/guardians participated in the study. Participation in the study was voluntary and the children were free to withdraw from the study at any time. For confidentiality purposes, unique codes were assigned to each participant, which were used to identify them for the purposes of the study instead of their names. All the study records were kept at a secured local store by the principal investigator (PI). All the children with schistosomiasis or STH were treated using PZQ (40 mg/kg) and/or ALB [21]. Any side-effects related to the PZQ treatment were monitored and managed by health professionals (nurses, medical doctors) who were part of the team.

### Data analysis

Data were double entered into a Microsoft Excel sheet, cleaned, and exported to Stata version 15 (Stata statistical software 2017; StataCorp, College Station, TX). The focus of the analysis was to determine the prevalence of STH, *S. haematobium* and *S. mansoni* based on the KK technique and urine filtration technique variables summarized as proportions, median or mean ± SD. Chi-square or Fisher’s exact tests were used to compare frequencies/proportions/categorical variables, whereas continuous variables were compared using a* t*-test. For *S. mansoni* and STH eggs, arithmetic mean egg counts were calculated from the counts of two KK smears and multiplied by 24 to obtain an individual’s number of eggs per gram of faeces. The mean egg counts for *S. mansoni* and STH were compared between sex and age groups using either* t*-test (two groups) or ANOVA (for more than two groups). The intensity of infection was categorized according to WHO criteria as low, moderate or heavy as follows: for *S. mansoni*, 1–99 eggs per gram of faeces (epg), 100–399 epg and ≥ 400 epg, respectively; for hookworm, 1–1999, 2000–3999 and ≥ 4000 epg, respectively; for *T. trichiura*, 1–999, 1000–9999 and ≥ 10,000, respectively; for *A. lumbricoides*, 1–4999, 5000–49,999 and ≥ 50,000, respectively [21]. For *S. haematobium*, infection intensities were classified into two categories as per WHO recommendations [22]: light infection (< 50 eggs/10 ml of urine) and heavy infection (≥ 50 eggs/10 ml of urine). The geometric mean egg output for each helminth species included in the study was estimated based on infected children only.

The observed combined prevalence (results from all wards/schools involved in the study to calculate the prevalence of schistosomiasis) of *S. haematobium* and *S. mansoni* infection was used to categorize implementation units (wards) for treatment strategies based on WHO prevalence thresholds as follows: high risk area (prevalence ≥ 50%), moderate risk area (prevalence ≥ 10% and < 50%), and low risk area (prevalence 1% and < 10%)[23]. The combined prevalence of STH was also used to categorize implementation units for treatment strategies as follows: low risk areas (> 20% and < 50%) and high-risk area (prevalence ≥ 50%) [10].

## Results

### Demographic information on school children

A total of 8698 SAC, comprising 4375 (50.3%) girls and 4323 (49.7%) boys, from 145 schools participated in the study. The mean age was 11.1 ± 1.6 years.

### Prevalences and intensities of schistosomiasis

The overall prevalence of *S. mansoni* across all districts was 8.7% [95% confidence interval (95%CI) 8.1–9.3], with prevalence higher for male than female participants (9.4% versus 8.1%, respectively, *P* = 0.03). However, the prevalence of the *S. mansoni* infection did not differ by age group (*P* = 0.48) (Table 1). The overall geometric mean number of eggs per gram of faeces was 166.56 epg, with males having the highest mean (25.1 epg versus 36.01 epg for females, *t* = − 2.4988, *P* = 0.03). No difference in mean eggs per gram of faeces was observed by age group (*t* = 0.6880, *P* = 0.49). In total, 36.4%, 41.6% and 21.9% of the SAC infected by *S. mansoni* had light, moderate and heavy intensity infections, respectively.

**Table 1:** Prevalence of *Schistosoma mansoni* and *Schistosoma haematobium* in north-western Tanzania stratified by age and sex of the study participants

Variable	*S. mansoni* infection		Chi-2, *P*-value	*S. haematobium* infection		Chi-2, *P*-value
Sex	Infected	Uninfected		Infected	Uninfected	
Male	9.4% (402)	90.6% (3891)	χ^2^(1) = 4.6544, *P* = 0.03	6.9% (298)	93.1% (4025)	χ^2^(1) = 10.8264, *P* = 0.001
Female	8.1% (350)	91.9% (3995)		5.2% (228)	94.8% (4147)	
Age groups in years						
9–10	8.4% (266)	91.5% (2890)	χ^2^(1) = 0.4812, *P* = 0.48	4.6% (145)	95.4% (3938)	χ^2^(1) = 19.6664, *P* = 0.001
11–13	8.9% (486)	91.1% (4096)		6.9% (381)	93.1% (5134)	

The overall prevalence of *S. haematobium* was 6.1% (95%CI 5.5–6.5). Male participants had the highest prevalence (males versus females, 6.9% versus 5.2%, χ^2^ = 10.8264, *P* = 0.001) (Table 1). The oldest age group had the highest prevalence (oldest versus youngest, 6.9% versus 4.6%, χ^2^ = 19.6664, *P* = 0.001). The overall geometric mean eggs/10 ml of urine was 19.05 eggs/10 ml (95%CI 16.7–21.6). Male participants had the highest geometric mean eggs/10 ml (males versus females, 3.8 versus 2.4 eggs/10 ml urine,* t* = − 2.6829, *P* = 0.01). Similar to prevalence of *S. haematobium*, the oldest age group (11–13 years) had a higher geometric mean eggs/10 ml of urine than the younger age group (9–10 years) (oldest versus youngest, 3.7 versus 2.0 eggs/10 ml of urine,* t* = − 3.1386, *P* = 0.002). Overall, 71.7% and 28.3% of the participants had light and heavy intensity infections, respectively. The overall prevalence of microhaematuria was 7.3% (95%CI 6.7–7.8), with males having a higher prevalence than females (8.4% versus 6.2%, respectively, *P* < 0.001).

### Prevalence and intensities of soil-transmitted helminths

The overall prevalence of hookworms was 6.2% (95%CI 5.6–6.6). There was no difference in prevalence according to sex (females versus males, 6.1% versus 6.3%, *P* = 0.2) (Table 2). High prevalence was observed in the older age group compared to the youngest age group (7.2% versus 4.5%, χ^2^ = 24.5476, *P* = 0.001). The overall geometric mean eggs per gram of faeces was 153.7 epg (95%CI 129.2–182.9), with no statistically significant difference between age groups (*t* =  − 1.1540, *P* = 0.2) or sexes (*t* = − 1.3486, *P* = 0.2). In total, 98.7% and 1.3% of the SAC had light and moderate intensities of infection, respectively.

**Table 2:** Prevalence of soil-transmitted helminths among school children in north-western Tanzania categorized by age and sex

Variable	Hookworm		Chi-2, *P*-value	*Ascaris lumbricoides*		Chi-2, *P*-value	*Trichuris trichiura*		Chi-2, *P*-value
	Positive	Negative		Positive	Negative		Positive	Negative	
**Sex**									
Female	6.1% (266)	93.8% (4079)	χ^2^(1) = 0.1715, *P* = 0.7	2.7% (118)	97.3% (4227)	χ^2^(1) = 0.8693, *P* = 0.35	1.4% (61)	98.6% (4284)	χ^2^(1) = 0.6207, *P* = 0.43
Male	6.3% (272)	93.6% (4020)		3.1 (131)	96.9% (4162)		1.2% (52)	97.7% (4241	
**Age groups (years)**									
9–10	4.5% (143)	95.5% (3013)	χ^2^(1) = 24.5476, *P* = 0.001	3.9% (122)	96.1% (3034)	χ^2^(1) = 17.1661, *P* = 0.001	1.7% (54)	98.3% (3102)	χ^2^(1) = 6.2511, *P* = 0.01
11–13	7.2% (315)	92.7% (5086)		2.3% (127)	97.6% (5355)		1.1% (59)	98.9% (5423)	

The overall prevalence of *A. lumbricoides* was 2.8% (95%CI 2.5–3.2) (Table 2), with no statistically significant difference between the sexes (*P* = 0.4). However, the youngest age group, 9–10 years, had the highest prevalence of *A. lumbricoides* (youngest versus oldest, 3.9% versus 2.3%, *P* = 0.001). The geometric mean eggs per gram of faeces was 670.2 epg (95%CI 555.8–808.2). The youngest age group (9–10 years) had the highest geometric mean eggs per gram of faeces (youngest versus oldest 72.5 versus 36.4 epg, *t* = 3.0971, *P* = 0.002). In total, 92.4% and 7.6% of the participants had light and moderate ascaris infection intensities, respectively.

The overall prevalence of *T. trichiura* was 1.3% (95%CI 0.1–1.5) (Table 2), and the youngest age group had the highest prevalence (youngest versus oldest, 1.7% versus 1.1%, *P* = 0.012). The overall geometric mean per gram of faeces was 143.5 epg (95%CI 11.98–171.8), with no statistically significant difference between age groups (*t* = − 0.0775, *P* = 0.9) or males and females (*t* = 0.1731, *P* = 0.9). Most participants had low intensity infections (97.4%), while only 0.8% had moderate and 1.6% heavy intensity infections.

### Prevalence of combined schistosomiasis, soil-transmitted helminths and endemicity status

Table 3 shows the overall combined prevalence of schistosomiasis (*S. haematobium* and *S. mansoni*) and the overall combined prevalence of STH species (hookworm, *A. lumbricoides* and *T. trichiura*) stratified by gender and district. The overall combined prevalence of schistosomiasis was 14.6% (95%CI 13.9–15.4) and ranged from 0 to 53%; males (16.2%) had the highest prevalence (13.1% for females) (*P* < 0.001). Significant differences in prevalence of schistosomiasis were noted between districts (*P* < 0.001), wards/sub-districts (*P* < 0.001) and schools (*P* < 0.001). Ukerewe district had the highest prevalence of schistosomiasis (53%) of all the districts. *Schistosoma mansoni* was common in Kigoma, Mwanza and Mara regions, whereas *S. haematobium* was common in Simiyu and Shinyanga regions.

**Table 3:** Overall combined prevalences of schistosomiasis (*Schistosoma haematobium* and *Schistosoma mansoni*) and soil-transmitted helminthiasis (*STH*) by district

Variables	Prevalence of any/combine schistosomiasis (95%CI)	Endemicity category	Prevalence of any/combined STH (95%CI)	Endemicity category
Gender				
Male	16.2% (15.1–15.3)	–	9.5% (8.6–10.4)	–
Female	13.1% (12.2–14.2)	–	9.1% (8.3–9.9)	–
District				
Bariadi	10% (7.1–13. 9)	Low	2% (0.8–4.3)	Low
Bariadi Town Council (TC)	23.7% (19.2–28.8)	Moderate	7.3% (4.9–10.9)	Low
Biharamulo	5.7% (3.5–8.9)	Low	39% (33.6–44.6)	Moderate
Bukoba	2.7% (1.3–5.3)	Low	38% (32.6–43.6)	Moderate
Bunda	22% (17.2–5.3)	Moderate	15.7% (11.9–20.3)	Low
Busega	23.7% (19.2–28.8)	Moderate	0.7% (0.1–2.6)	Low
Kahama Municipal Council (MC)	6.7% (4.3–10.1)	Low	6.7% (4.3–10.1)	Low
Karagwe	0.3% (0–2.3)	Low	0.3% (0–2.3)	Low
Kasulu	13.3% (9.9–17.6)	Low	0.3% (0.0 –2.3)	Low
Kibondo	29% (24.4–34.7)	Moderate	9% (6.2–12.8)	Low
Kigoma District Council (DC)	12.3% (9.1–16.6)	Moderate	6.7% (4.3–10.2)	Low
Kigoma MC	7.4% (4.9–10.9)	Low	1% (0.3–3.1)	Low
Kishapu	10.3% (7.3–14.3)	Low	0	–
Kwimba	6% (3.8–9.3)	Low	1.7% (0.6–3.9)	Low
Maswa	39% (33.6–44.6)	Moderate	9.7% (6.7–13.5)	Low
Meatu	20% (15.8–24.9)	Moderate	4.3% (2.5–7.3)	Low
Misenyi	10.7% (7.6–14.7)	Moderate	38% (32.6–43.6)	Moderate
Msalala	0.8% (0.2–3.2)	Low	8.3% (5.4–12.5)	Low
Muleba	22.3% (17.9–27.4)	Moderate	20% (15.8–24.9)	Low
Musoma DC	20.7% (16.4–25.6)	Moderate	4% (2.2–6.9)	Low
Musoma MC	13.3% (9.9–17.6)	Moderate	0.6% (0.0–2.6)	Low
Ngara	0.3% (0–2.3)	Low	8.3% (5.7–12.1)	Low
Rorya	32.3% (27.3–37.8)	Moderate	1.7% (0.6–3.9)	Low
Sengerema	17.3% (13.4–22.1)	Moderate	6.7% (4.3–10.1%)	Low
Serengeti	8.3% (2.0–6.5)	Low	8.3% (2.0–6.5)	Low
Shinyanga	10% (7.1–13.9)	Low	4.3% (2.5–7.3)	Low
Tarime	1.7% (0.6–3.9)	Low	9.7% (6.7–13.5)	Low
Ukerewe	53% (47.3–58.6)	High	2.3% (1.1–4.8)	Low
Ushetu	6.3% (4.1–9.7)	Low	14% (10.5–18.4)	Low

95%*CI* 95% Confidence interval

The overall prevalence of any STH infection was 9.3% (95%CI 8.6–9.9), with no statistically significant difference between females and males (9.1% versus 9.5%, respectively, *P* = 0.56). STH were common in Kagera region and Kigoma region. Hookworm was also common in dry areas of Simiyu region and Shinyanga region.

**Fig. 2:**
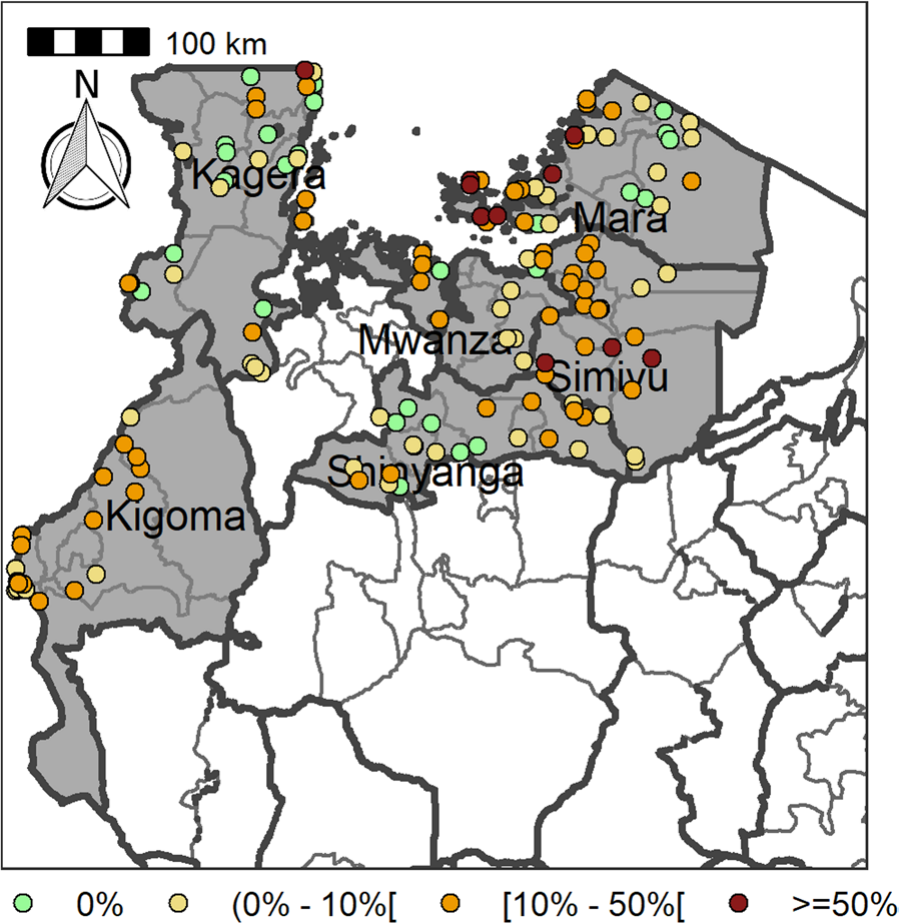
Prevalence of any schistosome by site/school. Map includes county and district boundaries

**Fig. 3:**
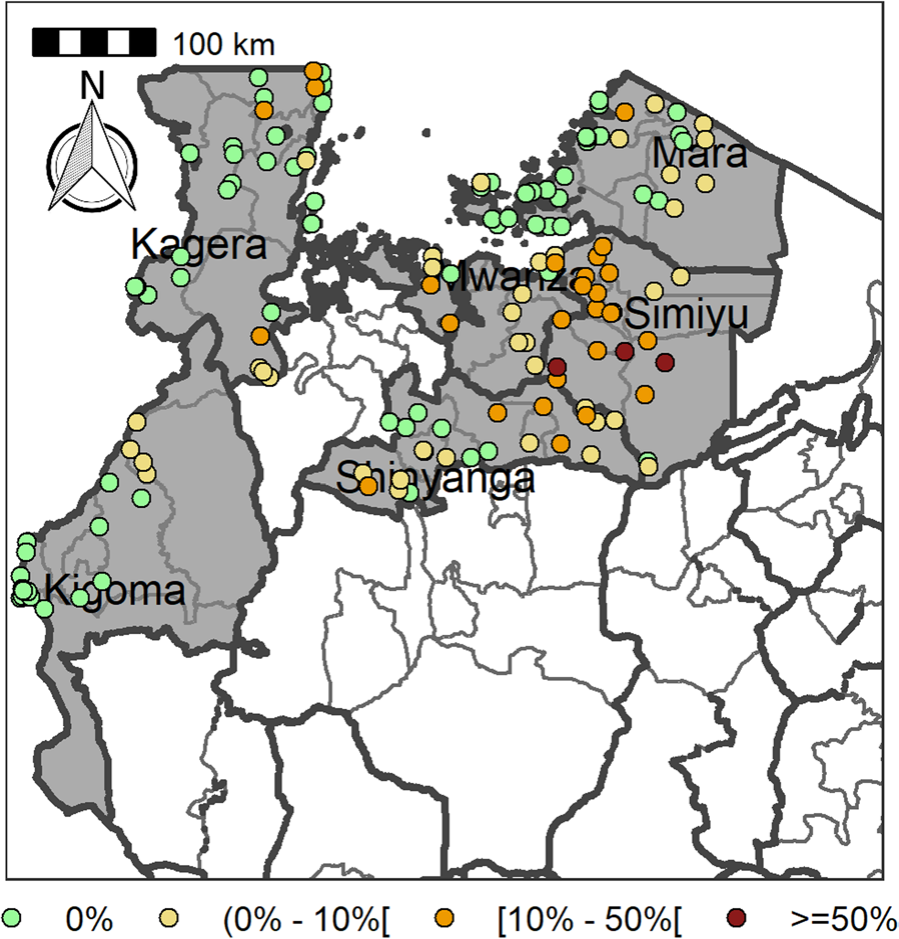
Prevalence of *Schistosoma haematobium* by site/school. Map includes county and district boundaries

**Fig. 4:**
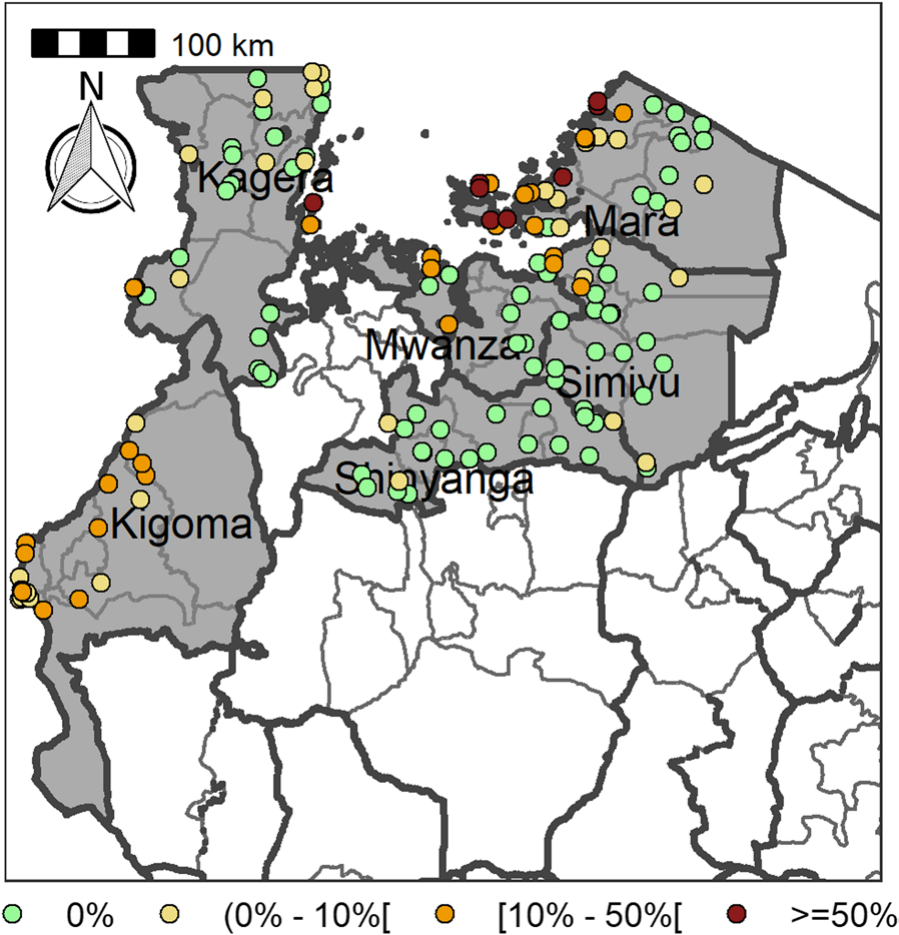
Prevalence of *Schistosoma mansoni* by site/school. Map includes county and district boundaries

**Fig. 5:**
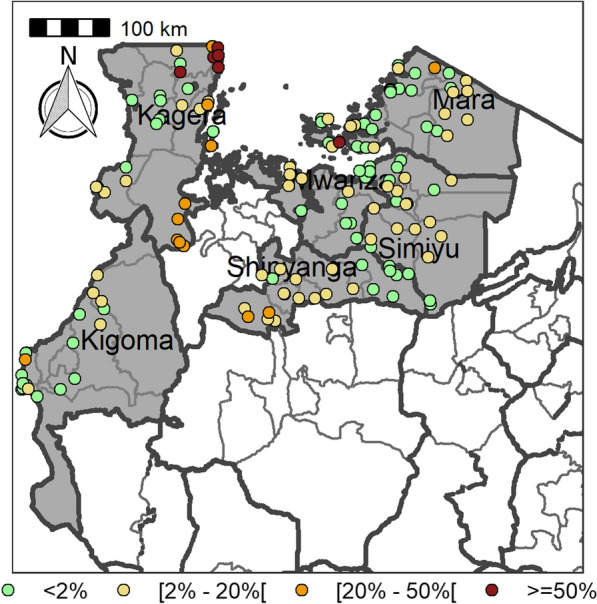
Prevalence of any soil-transmitted helminthiasis (STH) by site/school. Map includes county and district boundaries

**Fig. 6:**
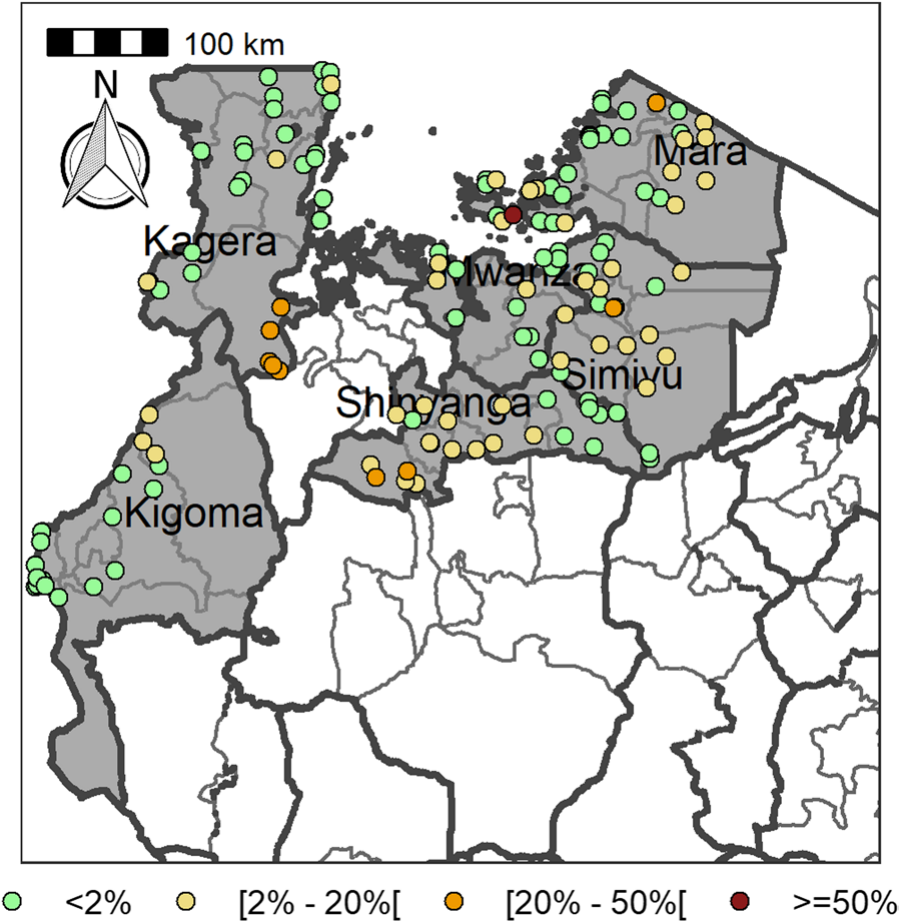
Prevalence of hookworm by site/school. Map includes county and district boundaries

**Fig. 7:**
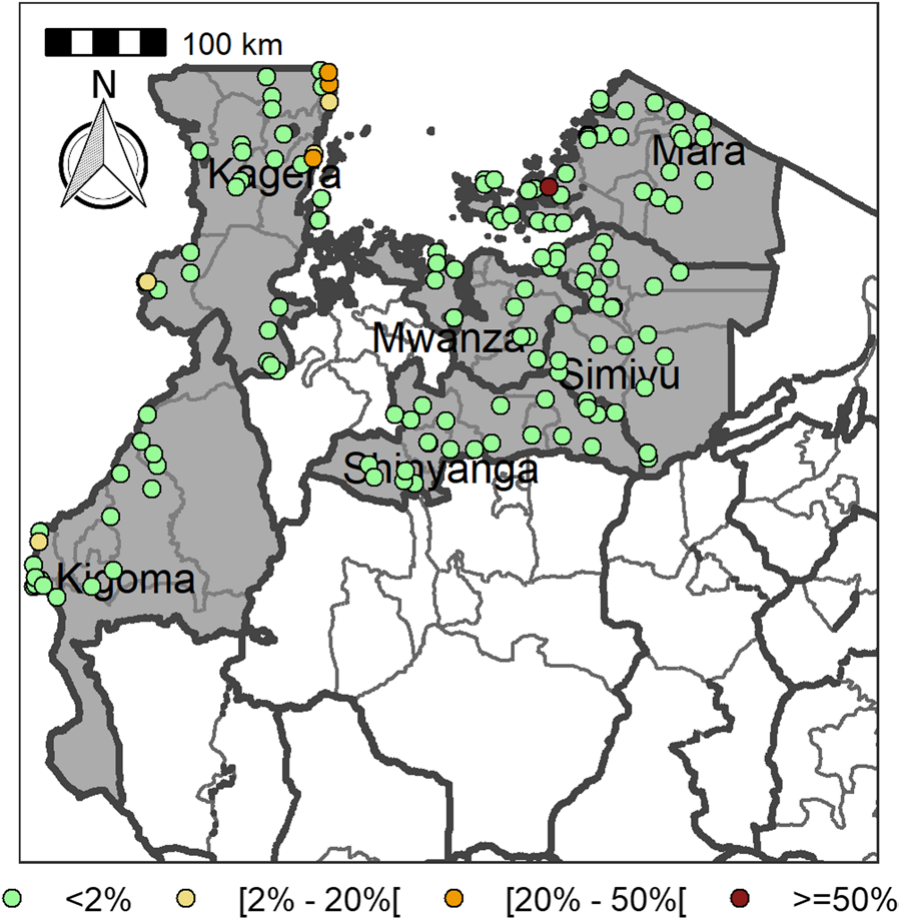
Prevalence of* Trichuris trichuris* by site/school. Map includes county and district boundaries

**Fig. 8:**
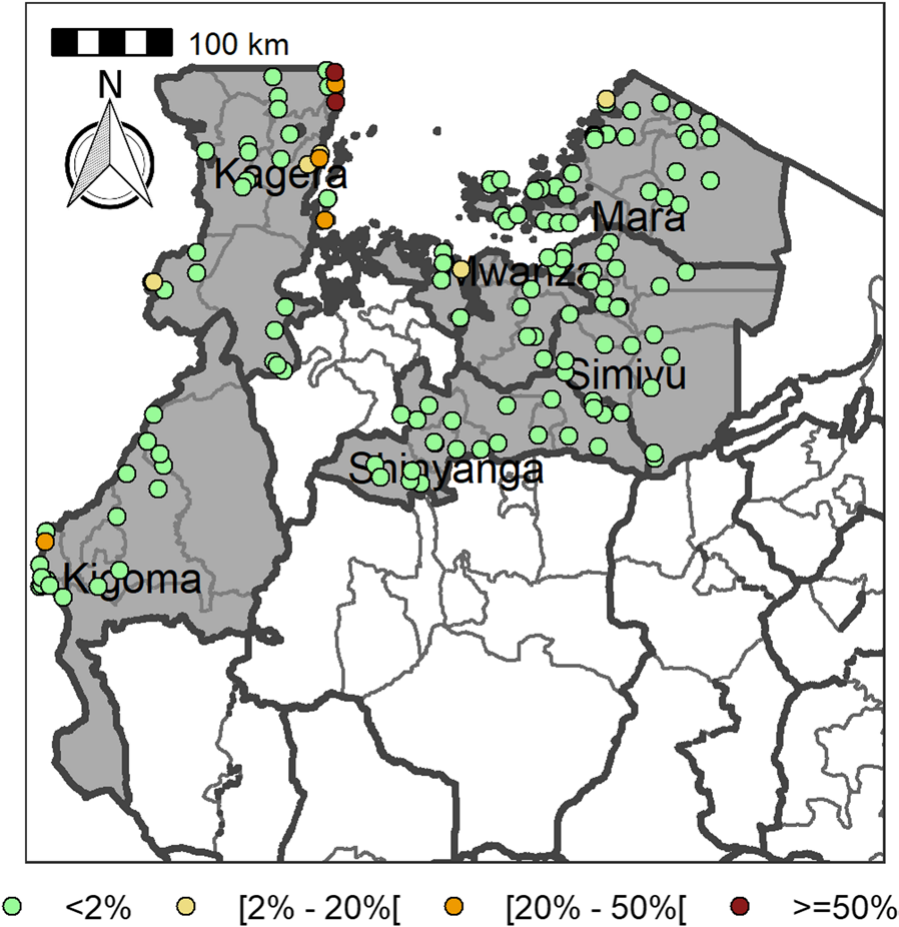
Prevalence of* Ascaris lumbricoides* by site/school. Map includes county and district boundaries

Table 4 summarizes the prevalence of any schistosome and STH species and endemicity status of each district and ward. The combined prevalence of schistosomiasis was used to categorize implementation units (districts and wards) for treatment strategies based on the WHO prevalence thresholds [schistosomiasis, high risk area (prevalence ≥ 50%), moderate risk area (prevalence ≥ 10% and < 50%), and low risk area (prevalence 1% to < 10%)]. The combined prevalence of STH was also used to categorize implementation units for treatment strategies as follows: high-risk areas (prevalence ≥ 50%) and low risk areas (≥ 20%). Figures 2, 3, 4, 5, 6, 7 and 8 show the prevalence and distribution of schistosome and STH species in the study areas.

**Table 4:** Overall combined prevalences of schistosomiasis (*Schistosoma haematobium* and *Schistosoma mansoni*) and STH by ward/sub–district

District	Ward	Prevalence of any/combine schistosomiasis	Endemicity category	Prevalence of any/combined STH	Endemicity category
Bariadi	Banemhi	8.3%	Low	3.3%	Low
	Mwasubuya	20%	Moderate	3.3%	Low
	Nkololo	5%	Low	1.7%	Low
	Sakwe	13.3%	Low	1.7%	Low
	Sapiwi	3.3%	Low	0	–
Bariadi TC	Guduwi	25%	Moderate	13.3%	Low
	Mhango	20%	Moderate	3.3%	Low
	Nyakabidi	46.7%	Moderate	3.3%	Low
	Somanda	1.7%	Low	0	–
Biharamulo	Kabindi	0	–	23.3%	Moderate
	Kaniha	5%	low	46.7%	Moderate
	Nemba	13.3%	Low	43.3%	Moderate
	Nyantakara	5%	low	40.8%	Moderate
Bukoba	Izimbya	0	–	0	
	Kishanje	0	–	51.7%	High
	Nyakato	0	–	58.3%	High
	Rubafu	8.3%	Low	71.7%	High
	Rukoma	5%	Low	8.3%	low
Bunda	Butimba	8.3%	Low	6.7%	Low
	Chitengule	26.7%	Moderate	0	–
	Hunyari	0	–	0	–
	Kisorya	75%	High	71.7%	High
	Mugeta	0	–	–	–
Busega	Mkula	13.3%	Moderate	0.8%	Low
	Mwamanyili	33.3%	Moderate	0	–
	Ngasamo	10%	Low	1.7%	Low
	Nyashimo	48.3%	Moderate	0	–
Kahama MC	Isagehe	5%	Low	0	–
	Kagonngwa	5%	Low	13.3%	Low
	Kinaga	6.7%	Low	6.7%	Low
	Nyandekwa	8.3%	Low	6.7%	Low
	Zangomera	8.3%	Low	6.7%	Low
Karagwe	Bugene	0	–	0	–
	Kamagambo	1.7%	Low	1.7%	Low
	Nyakahanga	0	–	0	–
	Rugu	0	–	0	–
Kasulu	Buhoro	16.7%	Moderate	0	–
	Kasulu	11.1%	Moderate	0	–
	Rungwe	1.7%	Low	1.7%	Low
Kibondo	Busagara	8.3%	Low	11.7%	Low
	Busunzu	31.7%	Moderate	0	–
	Kumsenga	13.3%	Low	5%	Low
	Mabamba	45%	Moderate	16.7%	Low
	Rugongwe	48%	Moderate	11.7%	Low
Kigoma DC	Kagunga	13.3%	Low	0	–
	Mwamgongo	28.3%	Moderate	31.7%	Moderate
	Simbo	13.3%	Low	1.7%	Low
	Ziwani	3.3%	Low	0	0
Kigoma MC	Bangwe	5%	Low	0	–
	Kibirizi	10%	Low	0	–
	Machinjioni	6.9%	Low	5.2%	Low
Kishapu DC	Kishapu	10%	Low	0	–
	Mwakipoya	5%	Low	0	–
	Songwa	13.3%	Low	0	–
	Talaga	13.3%	Low	0	–
Kwimba	Mwakilyabiti	6.7%	Low	1.7%	Low
	Mwan’alanga	6.7%	Low	1.7%	Low
	Ng’hungumalwa	5%	Low	0	0
	Ngula	5%	Low	3.3%	Low
	Nyamiti	6.7%	Low	1.7%	Low
Maswa	Badi	51.7%	High	10%	Low
	Ipililo	58.3%	High	13.3%	Low
	Jija	33.3%	Moderate	6.7%	Low
	Masela	25%	Moderate	1.7%	Low
	Zanzui	26.7%	Moderate	16.7%	Low
Meatu	Bukundi	3.3%	Low	1.7%	Low
	Isengwa	23.3%	Moderate	8.3%	Low
	Mwabuma	11.7%	Low	6.7%	Low
	Mwabuzo	6.7%	Low	0	–
	Sakasaka	55%	High	5%	Low
Misenyi	Ishunju	0	–	51.7%	High
	Kasambya	10%	Low	0	–
	Mushasha	0	–	98.3%	High
	Mutukula	0	–	11.7%	Low
	Kashenye	28.3%	Moderate	28.3%	Moderate
Msalala	Chela	0	–	3.3%	Low
	Isaka	0	–	6.7%	Low
	Mega	3.3%	Low	8.3%	Low
	Mwanase	0	–	15%	Low
Muleba	Ijumbi	0	–	11.7%	Low
	Ikuza	48.3%	Moderate	31.7%	Moderate
	Izigo	0	–	18.3%	Low
	Katoke	10%	Low	36.7%	Moderate
	Mazinga	53.3%	High	1.7%	Low
Ngara	Buririro	0	0	10%	Low
	Kabanga	0	0	20%	Low
	Murukulazo	1.7%	Low	3.3%	Low
Musoma DC	Bungwema	1.7%	Low	1.7%	Low
	Bukumi	16.7%	Moderate	13.3%	Low
	Makojo	20%	Moderate	3.3%	Low
	Musanja	6.7%	Low	1.7%	Low
	Suguti	58.3%	High	0	Low
Musoma MC	Kamunyonge	11.7%	Low	1.7%	Low
	Kwangwa	6.7%	Low	0	–
	Mshikamano	3.3%	Low	0	–
	Mwisenge	36.7%	Moderate	1.7%	Low
	Nyasho	8.3%	Low	0	–
Rorya	Kigunga	28.3%	Moderate	0	–
	Kisumwa	5%	Low	0	–
	Komuge	8.3%	Low	0	–
	Kyanga	60%	High	3.3%	Low
	Nyamagaro	60%	High	5%	Low
Sengerema	Chifunfu	21.7%	Moderate	6.7%	Low
	Igalula	20%	Moderate	1.7%	Low
	Kasongamilo	23.3%	Moderate	5%	Low
	Nyamatongo	0	–	13%	Low
Serengeti	Issenye	0	–	1.7%	Low
	Kisake	0	–	15%	Low
	Natta	3.3%	Low	8.3%	Low
	Nyamatare	5%	Low	6.7%	Low
	Standi Kuu	10%	Low	10%	Low
Shinyanga	Didia	0	Low	10%	Low
	Iselamaganga	5%	Low	3.3%	Low
	Lyabusalu	8.3%	Low	1.7%	Low
	Puni	3.3%	Low	0	–
	Samuye	33.3%	Moderate	6.7%	Low
Tarime	Binagi	0	–	1.7%	Low
	Bumera	3.3%	Low	36.7%	Moderate
	Kwihancha	3.3%	Low	5%	Low
	Matongo	0	–	1.7%	Low
	Muriba	1.7%	Low	3.3%	Low
Ukerewe	Bukiko	31.7%	Moderate	3.3%	Low
	Bukungu	61.7%	High	1.7%	Low
	Bwisya	76.7%	High	0	0
	Nansio	60%	High	1.7%	Low
	Ngoma	35%	High	5%	Low
Ushetu	Bulingwa	13.3%	Low	30%	Moderate
	Kisuke	10%	Low	26.7%	Moderate
	Mapamba	0	–	3.3%	Low
	Nyamilangano	3.3%	Low	3.3%	Low
	Ulewe	5%	Low	6.7%	Low

For abbreviations, see Table 3

### Prevalence and intensities of schistosomiasis (*S. mansoni* and* S. haematobium*) and soil-transmitted helminths for each school and ward/sub-district

Additional file 1: Tables S1-S58 and Fig. S1-S6 summarize the prevalence and intensities of each of the helminth species recorded for each school and sub-district included in the study. Overall, there was variation in the prevalence and intensity of schistosomiasis and STH between schools and sub-districts.

## Discussion

This study reports the distribution of schistosomiasis and STH prevalence in 29 districts of north-western Tanzania. At least one of the forms of schistosomiasis (urogenital or intestinal, which are caused by *Schistosoma haematobium* and *Schistosoma mansoni* respectively) occurred in all the regions of north-western Tanzania examined here. *Schistosoma mansoni* was observed to be endemic in districts bordering Lake Victoria to the south, west and east, and on an island in the lake. It was also endemic in a district bordering Lake Tanganyika and was found in schools/sub-districts located close to water bodies away from the lake. *Schistosoma mansoni* was a lesser problem in districts located beyond the shorelines of the large water bodies, particularly Lake Victoria and Lake Tanganyika, with prevalence declining as the distance from the lake basins increased; these results generally corroborate the findings of previous surveys [5].

*Schistosoma haematobium* was noted as a public health concern in districts located beyond Lake Victoria and Lake Tanganyika basins, with high prevalences recorded for Simiyu and Shinyanga regions. These findings corroborate results of previous mapping in the same regions [5, 24]. In general, the distributions of *S. mansoni* and *S. haematobium* in the north-western regions are related to the behaviour and ecological preferences of their intermediate host snails, i.e. *Bulinus* spp. for *S. haematobium* and *Biomphalaria* spp. for *S. mansoni* [25–27]. The ability of *Bulinus* snails to hibernate during the dry seasons, a characteristic not seen in *Biomphalaria* snails, means that they are able to colonize seasonal water bodies. Seasonal water bodies are commonly seen at the southern end of Lake Victoria, which can explain the high prevalence of *S. haematobium* there. In contrast, *Biomphalaria* snails prefer permanent freshwater bodies, and thus communities living along large freshwater bodies and on inland islands have high prevalences of *S. mansoni* [28]. Because of these characteristics, the transmission of *S. haematobium* is generally more widespread than that of *S. mansoni*, and it is also more seasonal, with highest transmission occurring after the rainy season when *Bulinus* snails are no longer dormant [25].

The STH tended to occur in areas where schistosomiasis occurs; however, prevalences in the western regions, and especially in the districts of Kagera region, were high compared to other districts. Hookworm was widely distributed and occurred in almost all regions at varying levels of prevalence. High prevalences of hookworm were recorded for ecological areas that experience higher temperatures, e.g. Ushetu and Maswa districts. Hookworm is known to be widespread in many areas with high temperatures due to the higher survival rates of its larval stages under these conditions [29]. *Ascaris lumbricoides* and *T. trichiura* were noted in areas with a wet climate, especially in Kagera and Kigoma regions. These two helminths are known to survive well in areas with a sub-tropical climate characterized by heavy rainfall and lower temperatures throughout the year [29].

We noted an overlapping distribution of schistosomiasis and STH in the six regions included in the study, which can be partly explained by their similarity with respect to certain conditions [30]. Putatively, these include the occurrence of wet soils due to high rainfall, average temperatures suitable for parasite survival, poor access of inhabitants to sanitation and hygiene facilities, and livelihood-related activities that predispose communities to these infections [30]. Livelihoods like farming in high risk endemic areas, and fishing and domestic and recreational activities in infested water bodies increase the risk and frequency of exposure of community members to contaminated water, and generally poor sanitation facilities result in environmental contamination with faeces. The co-occurrence of schistosomiasis and STH corroborates previous findings from these regions [29, 31].

### Implications of the current findings for MDA programmes

The geographical prevalences of schistosomiasis and STH in the current study have several implications for MDA in terms of planning, implementation, and evaluation with the aim of shrinking the schistosomiasis and STH map in Tanzania. The WHO have set criteria for schistosomiasis prevalence and morbidity control [32], with an ambitious goal of eliminating schistosomiasis as a public health problem (defined as < 1% of heavy intensity infections) in all endemic countries by 2030 [33]. Based on this criterion, morbidity control can be achieved if the prevalence of heavy intensity infections with any schistosome species is reduced to < 5% [32]. Our results indicate that the studied sub-districts had an overall prevalence of heavy intensity infections of 23% and 24% for *S. mansoni* and *S. haematobium*, respectively. The use of intensity of infection as an indicator for reaching the elimination stage is debatable, and has attracted different views [34]. To reach the elimination stage for schistosomiasis, the WHO has released new recommendations that MDA should be offered to either school children or community members based on the disease’s prevalence [23]. For communities living in areas with a prevalence of ≥ 10% of any *Schistosoma* spp., a single annual round of MDA using PZQ is recommended with at least ≥ 75% coverage for all age groups from 2 years of age to control morbidities [23]. For communities living in areas with a prevalence of < 10% of any *Schistosoma* species, the WHO recommends that (i) mass preventive chemotherapy should continue at the same frequency and be reduced at the interruption of transmission, and (ii) in areas that do not have access to mass preventive chemotherapy, a test-and-treat approach should be used instead [23]. To ensure wide coverage of treatment, the WHO recommends that health facilities should provide access to treatment with PZQ to control morbidity due to schistosomiasis in all infected individuals regardless of age [23]. However, the capacity of primary health facilities to provide diagnoses and treatment against schistosomiasis in sub-Saharan Africa is questionable [23]. Environmental interventions (including water engineering and focal snail control with molluscicides) and behavioural change interventions are also recommended to break the transmission of schistosomiasis in endemic areas [23]. Several sites included in the current study had different prevalences of schistosomiasis, which means that they fall within different categories of WHO recommended intervention. Thus, when planning for the next round of preventive chemotherapies, current local prevalence data should be reviewed for the national control programme and decisions based on the new treatment recommendations of the WHO [23].

Similarly, STH are targeted for elimination as a public health problem by 2030, defined by the WHO [35] in their revised neglected tropical disease roadmap for 2021–2030 as < 2% of moderate and heavy intensity STH infections. A large-scale MDA intervention is recommended when infection in SAC is ≥ 20% [35]. In communities with a prevalence of ≥ 50%, the WHO recommends treatment of all SAC, irrespective of whether or not they are enrolled in school, twice per year, and even three times per year if resources are available; in communities where prevalence is ≥ 20% but < 50%, SAC should be treated once a year [36]. The sub-districts studied here had an overall prevalence of heavy intensity infections for hookworm, *T. trichiura* and *A. lumbricoides* of < 1%, indicating that Tanzania is moving towards achieving WHO goals in eliminating STH as a public health problem [33]. However, given the variation in prevalence of the STHs, with hookworm being the most common species, and considering that adults are reservoirs of hookworm infection, it is recommended that the adult population should also be included in community-wide mass treatment programmes. School- and community-based MDA programmes have been shown to have a significant impact in controlling STH infections in endemic areas [10]. As STH infections are prevalent in areas characterized by limited access to safe water sources, sanitation and hygiene [10], integrated intervention measures are highly recommended [33]. Thus, to meet the elimination goals of the WHO, MDA campaigns must be supplemented with improved clean water supplies, sanitation, hygiene, and public health education that aims to change behaviours.

Based on the WHO criteria, the majority of the school/sub-districts included in the precision mapping survey had low prevalences (< 10%) for schistosomiasis and STH (< 20%) and moderate prevalences for schistosomiasis (between 10 and 49%) and STH (between ≥ 20% and 50%) in transmission areas. Thus, according to WHO recommendations, most of the schools located in these areas with moderate endemicity will require either a single annual round or a single round of treatment once every 2 years of ALB and PZQ. On the other hand, schools located in areas with low endemicity may not need annual MDA, but instead a single round of MDA every 2 years. To significantly reduce prevalence and interrupt transmission in areas classified as moderate risk it is important to intensify intervention measures in these areas in the future. Alternatively, a well-focused treatment strategy needs to be designed that is focused on schools/sub-districts in areas classified as moderate risk, instead of implementing the current approach which includes treatment in every school. However, for informed decision-making, the mapping of schistosomiasis and STH in all schools in an implementation unit (district or ward) is recommended.

### Limitations of the study

The parasitological results used to define the prevalences and intensities of infection were based on single urine and stool samples collected from the study participants. This may have led to the underestimation of the true prevalences and intensities of infections. In addition, the sampling strategy mainly targeted schistosomiasis, which may have resulted in the underestimation of the prevalence of STH. The cross-sectional nature of the study design meant that data on causality could not be collected. However, using this design we were able to describe the prevalence and intensities of the targeted infections. Despite these limitations, the findings reported here allow for the following conclusions and recommendations to be made.

## Conclusions

The present study provides data on the geographical distribution of schistosomiasis and STH, their co-endemicity, and prevalences at different levels of intensities of infection with any schistosome or STH species in 29 districts across six regions of north-western Tanzania. Current MDA strategies can be reviewed and updated on the basis of these results. As some areas no longer require MDA, scarce resources can be saved. A focused MDA strategy targeting schools/sub-districts where the prevalence of schistosomiasis and STH is ≥ 10% and ≥ 15%, respectively, as shown here, would support Tanzania’s control and elimination goals. To achieve these elimination goals, complementary/supplementary strategies including health education and the provision of safe water and adequate sanitary facilities should be implemented concurrently.

## Supplementary Information

**Additional file 1: Additional Tables S1–S58.** **Figure S1**. Geographical locations of schools included in the precision mapping in five districts of Simiyu region, north-western Tanzania. **Figure S2**. Geographical locations of schools included in the precision mapping in three districts of Mwanza region, north-western Tanzania. **Figure S3**. Geographical locations of schools included in the precision mapping in four districts of Kigoma region, western Tanzania. **Figure S4**. Geographical locations of schools included in the precision mapping in five districts of Shinyanga region, north-western Tanzania. **Figure S5**. Geographical locations of schools included in the precision mapping in five districts of Mara region, north-western Tanzania. **Figure S6**. Geographical locations of schools included in the precision mapping in five districts of Kagera region, north-western Tanzania.

## Data Availability

The datasets used and/or analysed during the current study are available from the corresponding author on reasonable request.
