# Supplementary material for: Precision and geographical prevalence mapping of schistosomiasis and soil-transmitted helminthiasis among school-aged children in selected districts of north-western Tanzania
Source: Parasit Vectors. 2022 Dec 29;15:492. doi: 10.1186/s13071-022-05547-6 (PMC9801628; doi:10.1186/s13071-022-05547-6)
Supplement: Supplementary file 1 — Additional file 1: Additional Tables S1–S58. Figure S1. Geographical locations of schools included in the precision mapping in five districts of Simiyu region, north-western Tanzania. Figure S2. Geographical locations of schools included in the precision mapping in three districts of Mwanza region, north-western Tanzania. Figure S3. Geographical locations of schools included in the precision mapping in four districts of Kigoma region, western Tanzania. Figure S4. Geographical locations of schools included in the precision mapping in five districts of Shinyanga region, north-western Tanzania. Figure S5. Geographical locations of schools included in the precision mapping in five districts of Mara region, north-western Tanzania. Figure S6. Geographical locations of schools included in the precision mapping in five districts of Kagera region, north-western Tanzania. [file 13071_2022_5547_MOESM1_ESM.docx]

**Prevalence and intensities of schistosomiasis (*S. mansoni* and *S. haematobium*) and soil-transmitted helminths for each school and wards**

The prevalence and intensities of each of the helminth species included in the study for each school and district are shown in the tables below.

**Simiyu region**

**Bariadi district council**

Table 1: Prevalence and intensities of schistosomiasis at Bariadi district council

| **Ward** | **School** | **N** | ***S. mansoni*** | | | | ***S. haematobium*** | | |
| --- | --- | --- | --- | --- | --- | --- | --- | --- | --- |
|  |  |  | P | L | M | H | P | Low | Heavy |
| Banemhi | Kilabela A | 60 | 1(1.7%) | 1 (100%) | 0(0.0%) | 0(0.0%) | 5 (8.3%) | 5(100%) | 0 (100%) |
| Mwasumbuya | Mwasubuya B | 60 | 6 (10%) | 0 (0.0%) | 3(50%) | 3(50%) | 6 (10%) | 5(83.3%) | 1 (16.7%) |
| Nkololo | Mwabalizi | 60 | 0 (0%) | 0 (0.0%) | 0(0.0%) | 0(0.0%) | 3 (5%) | 3 (100%) | 0 (0.0%) |
| Sakwe | Mwangimu | 60 | 0 (0%) | 0 (0.0%) | 0(0.0%) | 0(0.0%) | 8(13.3%) | 8 (100%) | 0 (0.0%) |
| Sapiwi | Igegu | 60 | 0 (0%) | 0 (0.0%) | 0(0.0%) | 0(0.0%) | 2 (3.3%) | 2 (100%) | 0 (0.0%) |

**Key:** P=Prevalence, L=low, M=Moderate, H=Heavy

Table 2: Prevalence and intensity of soil-transmitted helminths at Bariadi district council

| **Ward** | **School** | **N** | ***A. lumbricoides*** | | | | ***T. trichiura*** | | | | ***Hookworm*** | | | |
| --- | --- | --- | --- | --- | --- | --- | --- | --- | --- | --- | --- | --- | --- | --- |
|  |  |  | P | L | M | H | P | L | M | H | P | L | M | H |
| Banemhi | Kilabela A | 60 | 0 | 0 | 0 | 0 | 0 | 0 | 0 | 0 | 3  3.3% | 100% | 0 | 0 |
| Mwasumbuya | Mwasubuya B | 60 | 0 | 0 | 0 | 0 | 0 | 0 | 0 | 0 | 2  3.3% | 100% | 0 | 0 |
| Nkololo | Mwabalizi | 60 | 0 | 0 | 0 | 0 | 0 | 0 | 0 | 0 | 0 | 0 | 0 | 0 |
| Sakwe | Mwangimu | 60 | 0 | 0 | 0 | 0 | 0 | 0 | 0 | 0 | 1  1.7% | 100% | 0 | 0 |
| Sapiwi | Igegu | 60 | 0 | 0 | 0 | 0 | 0 | 0 | 0 | 0 | 0 | 0 | 0 | 0 |

**Key:** P=Prevalence, L=Low, M=Moderate, H=Heavy

**Bariadi Town Council**

Table 3: Prevalence and intensities of *schistosomiasis* at Bariadi town council

| **Ward** | **School** | **N** | ***S. mansoni*** | | | | ***S. haematobium*** | | |
| --- | --- | --- | --- | --- | --- | --- | --- | --- | --- |
|  |  |  | P | L | M | H | P | Low | Heavy |
| Guduwi | Ditima | 60 | 0 | 0 | 0 | 0 | 16(26.7%) | 15(93.8%) | 1(6.2%) |
| Mhango | Shimbale | 60 | 0 | 0 | 0 | 0 | 12(20%) | 10(83.3%) | 2(16.7%) |
| Nyakabindi | Mwakibuga B | 60 | 0 | 0 | 0 | 0 | 28(46.7%) | 23(82.1%) | 5(17.9%) |
| Somanda | Nyaumata | 60 | 0 | 0 | 0 | 0 | 1(1.7%) | 1(100%) | 0 |
| Guduwi | Guduwi Mlimani | 60 | 0 | 0 | 0 | 0 | 14(23.3%) | 14(100%) | 0 |

**Key:** P=Prevalence, L=low, M=Moderate, H=Heavy

Table 4: Prevalence and intensity of soil-transmitted helminths at Bariadi town council

| **Ward** | **School** | **N** | ***A. lumbricoides*** | | | | ***T. trichiura*** | | | | ***Hookworm*** | | | |
| --- | --- | --- | --- | --- | --- | --- | --- | --- | --- | --- | --- | --- | --- | --- |
|  |  |  | P | L | M | H | P | L | M | H | P | L | M | H |
| Guduwi | Ditima | 60 | 0 | 0 | 0 | 0 | 0 | 0 | 0 | 0 | 2(3.3%) | 100% | 0 | 0 |
| Mhango | Shimbale | 60 | 0 | 0 | 0 | 0 | 0 | 0 | 0 | 0 | 2(3.3%) | 100% | 0 | 0 |
| Nyakabindi | Mwakibuga B | 60 | 0 | 0 | 0 | 0 | 0 | 0 | 0 | 0 | 4(6.7%) | 100% | 0 | 0 |
| Somanda | Nyaumata | 60 | 0 | 0 | 0 | 0 | 0 | 0 | 0 | 0 | 0 | 0 | 0 | 0 |
| Guduwi | Guduwi mlimani | 60 | 0 | 0 | 0 | 0 | 0 | 0 | 0 | 0 | 14(23.3%) | 100% | 0 | 0 |

**Key:** P=Prevalence, L=Low, M=Moderate, H=Heavy

**Busega district**

Table 5: Prevalence and intensities of schistosomiasis at Busega district council

| **Ward** | **School** | **N** | ***S. mansoni*** | | | | ***S. haematobium*** | | |
| --- | --- | --- | --- | --- | --- | --- | --- | --- | --- |
|  |  |  | P | L | M | H | P | Low | Heavy |
| Mkula | Kijireshi | 60 | 1(1.7%) | 100% | 0 | 0 | 9(15%) | 88.9% | 11.1% |
| Mwamanyili | Mwanangi | 60 | 13(21.7%) | 4(30.7%) | 3(23.1%) | 6(46.2%) | 8(3.3%) | 87.5% | 12.5% |
| Ngasamo | Sanga | 60 | 1 (1.7%) | 100% | 0 | 0 | 6(10%) | 83.3% | 16.7% |
| Nyashimo | Bulima | 60 | 28(46.7%) | 9(32.1%) | 16(57.1% | 3(10.7%) | 2(3.3%) | 100% | 0 |
| Mkula | Ng’wanhale | 60 | 0 | 0 | 0 | 0 | 6(10%) | 100% | 0 |

**Key:** P=Prevalence, L=Low, M=Moderate, H=Heavy

Table 6: Prevalence and intensity of soil-transmitted helminths at Busega district council

| **Ward** | **School** | **N** | ***A. lumbricoides*** | | | | ***T. trichiura*** | | | | ***Hookworm*** | | | |
| --- | --- | --- | --- | --- | --- | --- | --- | --- | --- | --- | --- | --- | --- | --- |
|  |  |  | P | L | M | H | P | L | M | H | P | L | M | H |
| Mkula | Kijireshi | 60 | 0 | 0 | 0 | 0 | 0 | 0 | 0 | 0 | 1(1.7%) | 100% | 0 | 0 |
| Mwamanyili | Mwanangi | 60 | 0 | 0 | 0 | 0 | 0 | 0 | 0 | 0 | 0 | 0 | 0 | 0 |
| Ngasamo | Sanga | 60 | 0 | 0 | 0 | 0 | 0 | 0 | 0 | 0 | 1(1.7%) | 100% | 0 | 0 |
| Nyashimo | Bulima | 60 | 0 | 0 | 0 | 0 | 0 | 0 | 0 | 0 | 0 | 0 | 0 | 0 |
| Mkula | Ng’wanhale | 60 | 0 | 0 | 0 | 0 | 0 | 0 | 0 | 0 | 0 | 0 | 0 | 0 |

**Key:** P=Prevalence, L=Low, M=Moderate, H=Heavy

**Maswa districts**

Table 7: Prevalence and intensities of schistosomiasis at Maswa district council

| **Ward** | **School** | **N** | ***S. mansoni*** | | | | ***S. haematobium*** | | |
| --- | --- | --- | --- | --- | --- | --- | --- | --- | --- |
|  |  |  | P | L | M | H | P | Low | Heavy |
| Badi | Bukangilija | 60 | 0 | 0 | 0 | 0 | 31(51.7%) | 48.4% | 51.6% |
| Ipililo | Ipililo | 60 | 0 | 0 | 0 | 0 | 35(58.3%) | 54.3% | 45.7% |
| Jija | Jija A &B | 60 | 0 | 0 | 0 | 0 | 20(33.3%) | 45% | 55% |
| Zanzui | Malita | 60 | 0 | 0 | 0 | 0 | 16(26.7%) | 37.5% | 62.5% |
| Masela | Wigelekelo | 60 | 0 | 0 | 0 | 0 | 15(25%) | 40% | 60% |

**Key:** P=Prevalence, L=Low, M=Moderate, H=Heavy

Table 8: Prevalence and intensity of soil-transmitted helminths at Maswa district council

| **Ward** | **School** | **N** | ***A. lumbricoides*** | | | | ***T. trichiura*** | | | | ***Hookworm*** | | | |
| --- | --- | --- | --- | --- | --- | --- | --- | --- | --- | --- | --- | --- | --- | --- |
|  |  |  | P | L | M | H | P | L | M | H | P | L | M | H |
| Badi | Bukangilija | 60 | 0 | 0 | 0 | 0 | 0 | 0 | 0 | 0 | 6(10%) | 100% | 0 | 0 |
| Ipililo | Ipililo | 60 | 0 | 0 | 0 | 0 | 0 | 0 | 0 | 0 | 8(13%) | 100% | 0 | 0 |
| Jija | Jija A&B | 60 | 0 | 0 | 0 | 0 | 0 | 0 | 0 | 0 | 4(6.7%) | 100% | 0 | 0 |
| Zanzui | Malita | 60 | 0 | 0 | 0 | 0 | 0 | 0 | 0 | 0 | 10(16.7%) | 100% | 0 | 0 |
| Masela | Wigelekelo | 60 | 0 | 0 | 0 | 0 | 0 | 0 | 0 | 0 | 1(1.7%) | 100% | 0 | 0 |

**Key:** P=Prevalence, L=Low, M=Moderate, H=Heavy

**Meatu district council**

Table 9: Prevalence and intensities of schistosomiasis at Meatu district council

| **Ward** | **School** | **N** | ***S. mansoni*** | | | | ***S. haematobium*** | | |
| --- | --- | --- | --- | --- | --- | --- | --- | --- | --- |
|  |  |  | P | L | M | H | P | Low | Heavy |
| Bukundi | Bukundi | 60 | 2(3.3%) | 0 | 0 | 0 | 0 | 0 | 0 |
| Isengwa | Semu | 60 | 0 | 0 | 0 | 0 | 14 (23.3%) | 42.9% | 57.1% |
| Mwabuma | Mwabuma | 60 | 0 | 0 | 0 | 0 | 7(11.7%) | 0 | 100% |
| Mwabuzo | Mwangikulu | 60 | 0 | 0 | 0 | 0 | 4(6.7%) | 50% | 50% |
| Sakaasaka | Sakasaka | 60 | 0 | 0 | 0 | 0 | 33(55%) | 42.4% | 57.6% |

**Key:** P=Prevalence, L=Low, M=Moderate, H=Heavy

Table 10: Prevalence and intensity of soil-transmitted helminths at Meatu district council

| **Ward** | **School** | **N** | ***A. lumbricoides*** | | | | ***T. trichiura*** | | | | ***Hookworm*** | | | |
| --- | --- | --- | --- | --- | --- | --- | --- | --- | --- | --- | --- | --- | --- | --- |
|  |  |  | P | L | M | H | P | L | M | H | P | L | M | H |
| Bukundi | Bukundi | 60 | 0 | 0 | 0 | 0 | 0 | 0 | 0 | 0 | 1(1.7%) | 100% | 0 | 0 |
| Isengwa | Semu | 60 | 0 | 0 | 0 | 0 | 0 | 0 | 0 | 0 | 5(8.3%) | 100% | 0 | 0 |
| Mwabuma | Mwabuma | 60 | 0 | 0 | 0 | 0 | 0 | 0 | 0 | 0 | 4(6.7%) | 100% | 0 | 0 |
| Mwabuzo | Mwangikulu | 60 | 0 | 0 | 0 | 0 | 0 | 0 | 0 | 0 | 0 | 0 | 0 | 0 |
| Sakasaka | Sakasaka | 60 | 0 | 0 | 0 | 0 | 0 | 0 | 0 | 0 | 3(15%) | 100% | 0 | 0 |

**Key:** P=Prevalence, L=Low, M=Moderate, H=Heavy


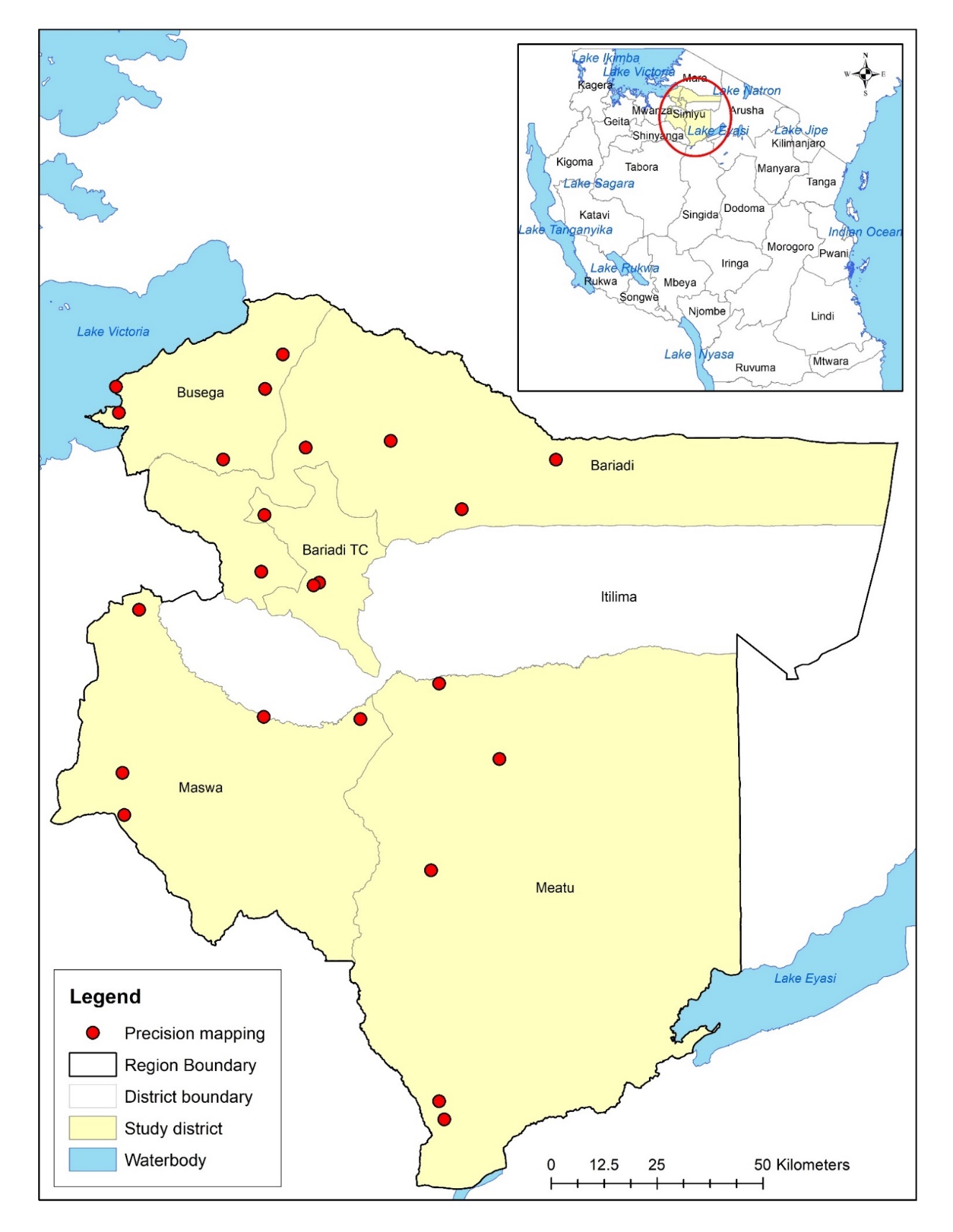


Figure 1: Geographical location of school involved in precision mapping in five districts of Simiyu region, north-western Tanzania

**Mwanza region**

**Ukerewe district council**

Table 11: Prevalence and intensities of schistosomiasis at Ukerewe district council

| **Ward** | **School** | **N** | ***S. mansoni*** | | | | ***S. haematobium*** | | |
| --- | --- | --- | --- | --- | --- | --- | --- | --- | --- |
|  |  |  | P | L | M | H | P | Low | Heavy |
| Bukiko | Bukiko | 60 | 19(31.7%) | 26.3% | 63.2% | 10.5% | 0 | 0 | 0 |
| Bukungu | Bukungu | 60 | 37(61.7%) | 24.3% | 40.5% | 35.1% | 1(1.7%) | 100% | 0 |
| Bwisya | Bwisya | 60 | 46(76.7%) | 15% | 47.8% | 36.9% | 0 | 0 | 0 |
| Nansio | Nansio | 60 | 36(60%) | 33.3% | 44.4% | 22.2% | 0 | 0 | 0 |
| Ngoma | Nantaare | 60 | 21(35%) | 61.9% | 28.6% | 9.5% | 0 | 0 | 0 |

**Key:** P=Prevalence, L=Low, M=Moderate, H=Heavy

Table 12: Prevalence and intensity of soil-transmitted helminths at Ukerewe district council

| **Ward** | **School** | **N** | ***A. lumbricoides*** | | | | ***T. trichiura*** | | | | ***Hookworm*** | | | |
| --- | --- | --- | --- | --- | --- | --- | --- | --- | --- | --- | --- | --- | --- | --- |
|  |  |  | P | L | M | H | P | L | M | H | P | L | M | H |
| Bukiko | Bukiko | 60 | 1(1.7%) | 100% | 0 | 0 | 0 | 0 | 0 | 0 | 2(3.3%) | 100% | 0 | 0 |
| Bukungu | Bukungu | 60 | 0 | 0 | 0 | 0 | 0 | 0 | 0 | 0 | 1(1.7%) | 100% | 0 | 0 |
| Bwisya | Bwisya | 60 | 0 | 0 | 0 | 0 | 0 | 0 | 0 | 0 | 0 | 0 | 0 | 0 |
| Nansio | Nansio | 60 | 0 | 0 | 0 | 0 | 0 | 0 | 0 | 0 | 1(1.7%) | 100% | 0 | 0 |
| Ngoma | Nantare | 60 | 0 | 0 | 0 | 0 | 0 | 0 | 0 | 0 | 3(5%) | 100% | 0 | 0 |

**Key:** P=Prevalence, L=Low, M=Moderate, H=Heavy

**Sengerema district council**

Table 13: Prevalence and intensities of schistosomiasis at Sengerema district council

| **Ward** | **School** | **N** | ***S. mansoni*** | | | | ***S. haematobium*** | | |
| --- | --- | --- | --- | --- | --- | --- | --- | --- | --- |
|  |  |  | P | L | M | H | P | Low | Heavy |
| Kasongamelo | Bulunga | 60 | 0 | 0 | 0 | 0 | 14(23.3% | 50% | 50% |
| Chifunfu | Chifunfu | 60 | 12(20%) | 50% | 33.3% | 16% | 3(5%) | 100% | 0 |
| Igalula | Chikomero | 60 | 7(11.7%) | 57.1% | 28.6% | 14.3% | 6(10%) | 100% | 0 |
| Chifunfu | Nyakahako | 60 | 12(26%) | 41.7% | 58.3% | 0 | 1(1.7%) | 100% | 0 |
| Nyamatongo | Nyamatongo | 60 | 0 | 0 | 0 | 0 | 0 | 0 | 0 |

**Key:** P=Prevalence, L=Low, M=Moderate, H=Heavy

Table 14: Prevalence and intensity of soil-transmitted helminths at Sengerema district council

| **Ward** | **School** | **N** | ***A. lumbricoides*** | | | | ***T. trichiura*** | | | | ***Hookworm*** | | | |
| --- | --- | --- | --- | --- | --- | --- | --- | --- | --- | --- | --- | --- | --- | --- |
|  |  |  | P | L | M | H | P | L | M | H | P | L | M | H |
| Kasongamelo | Bulunga | 60 | 0 | 0 | 0 | 0 | 0 | 0 | 0 | 0 | 3(5%) | 100% | 0 | 0 |
| Chifunfu | Chifunfu | 60 | 0 | 0 | 0 | 0 | 0 | 0 | 0 | 0 | 1(1.7%) | 100% | 0 | 0 |
| Igalula | Chikomero | 60 | 0 | 0 | 0 | 0 | 0 | 0 | 0 | 0 | 1(1.7%) | 100% | 0 | 0 |
| Chifunfu | Nyakahako | 60 | 0 | 0 | 0 | 0 | 0 | 0 | 0 | 0 | 7(11.7%) | 100% | 0 | 0 |
| Nyamatongo | Nyamatongo | 60 | 8(13.3%) | 100% | 0 | 0 | 0 | 0 | 0 | 0 | 0 | 0 | 0 | 0 |

**Key:** P=Prevalence, L=Low, M=Moderate, H=Heavy

**Kwimba district council**

Table 15: Prevalence and intensities of schistosomiasis at Kwimba district council

| **Ward** | **School** | **N** | ***S. mansoni*** | | | | ***S. haematobium*** | | |
| --- | --- | --- | --- | --- | --- | --- | --- | --- | --- |
|  |  |  | P | L | M | H | P | Low | Heavy |
| Mwakilyambiti | Ng’wamakoye | 60 | 0 | 0 | 0 | 0 | 4(6.7% | 100% | 0 |
| Mwang’alanga | Mahiga | 60 | 0 | 0 | 0 | 0 | 4(6.7%) | 100% | 0 |
| Ng’hungumalwa | Hungumalwa | 60 | 0 | 0 | 0 | 0 | 3(5%) | 100% | 0 |
| Ngula | Nyang’hingi | 60 | 0 | 0 | 0 | 0 | 3(5%) | 100% | 0 |
| Nyamiti | Ibindo | 60 | 0 | 0 | 0 | 0 | 4(6.7%) | 100% | 0 |

**Key:** P=Prevalence, L=Low, M=Moderate, H=Heavy

Table 16: Prevalence and intensity of soil-transmitted helminths at Kwimba district council

| **Ward** | **School** | **N** | ***A. lumbricoides*** | | | | ***T. trichiura*** | | | | ***Hookworm*** | | | |
| --- | --- | --- | --- | --- | --- | --- | --- | --- | --- | --- | --- | --- | --- | --- |
|  |  |  | P | L | M | H | P | L | M | H | P | L | M | H |
| Mwakilyambiti | Ng’wamakoye | 60 | 0 | 0 | 0 | 0 | 0 | 0 | 0 | 0 | 1(1.7%) | 100% | 0 | 0 |
| Mwang’alanga | Mahiga | 60 | 0 | 0 | 0 | 0 | 0 | 0 | 0 | 0 | 1(1.7%) | 100% | 0 | 0 |
| Ng’hungumalwa | Hungumalwa | 60 | 0 | 0 | 0 | 0 | 0 | 0 | 0 | 0 | 0 | 0 | 0 | 0 |
| Ngula | Nyan’hingi | 60 | 0 | 0 | 0 | 0 | 0 | 0 | 0 | 0 | 2(3.3%) | 100% | 0 | 0 |
| Nyamiti | Ibindo | 60 | 0 | 0 | 0 | 0 | 0 | 0 | 0 | 0 | 1(1.7%) | 100% | 0 | 0 |

**Key:** P=Prevalence, L=Low, M=Moderate, H=Heavy


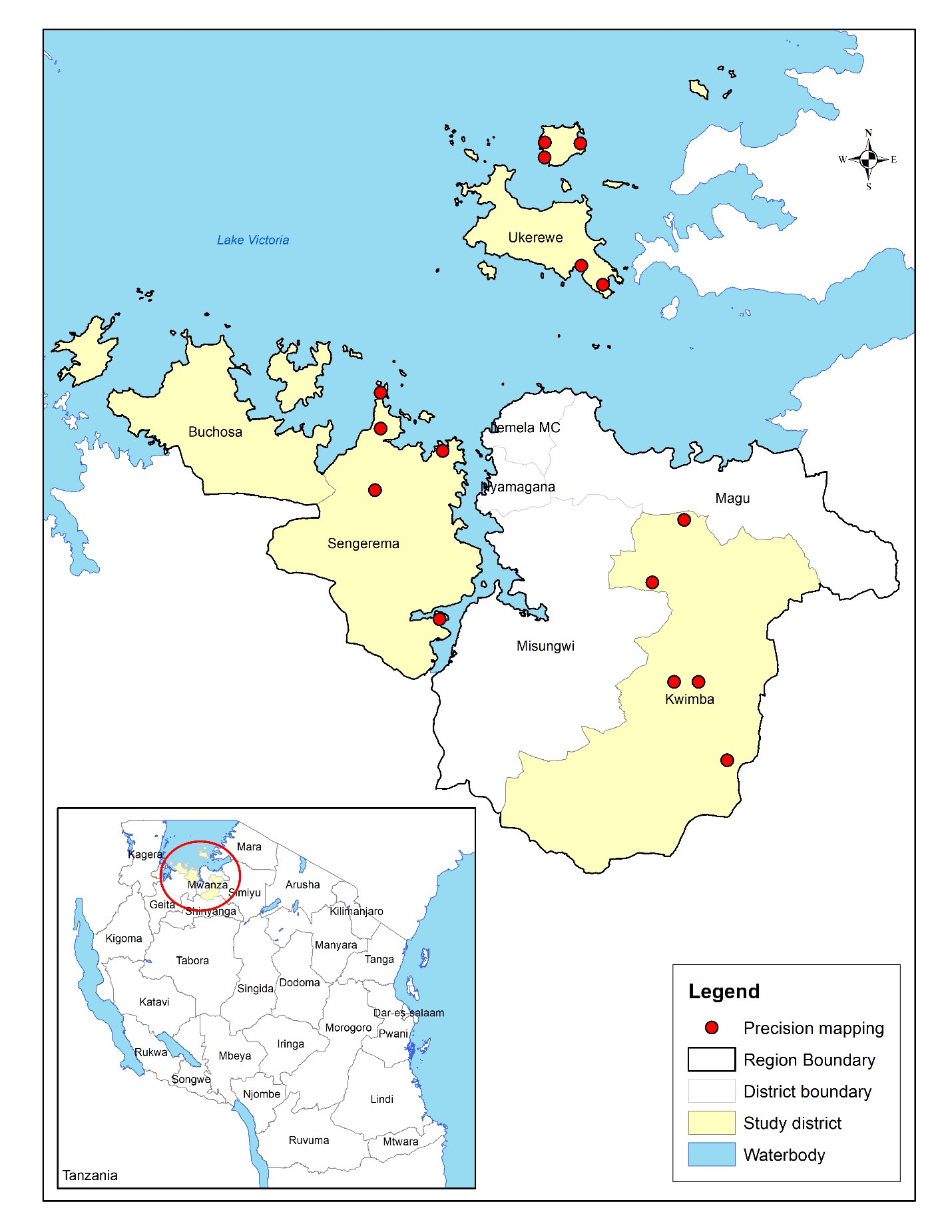


Figure 2: Geographical location of school involved in precision mapping in three districts of Mwanza, region, north-western Tanzania

**Kigoma region**

**Kasulu district council**

Table 17: Prevalence and intensities of schistosomiasis at Kasulu district council

| **Ward** | **School** | **N** | ***S. mansoni*** | | | | ***S. haematobium*** | | |
| --- | --- | --- | --- | --- | --- | --- | --- | --- | --- |
|  |  |  | P | L | M | H | P | Low | Heavy |
| Buhoro | Kibilizi | 60 | 10(16.7%) | 40% | 30% | 30% | 0 | 0 | 0 |
| Kasulu | Kigadye | 60 | 11(18.3%) | 55.5% | 45.5% | 0 | 0 | 0 | 0 |
| Kasulu | Kitibitibi | 60 | 8(13.3%) | 50% | 50% | 0 | 0 | 0 | 0 |
| Kasulu | Kumtundu | 60 | 1(1.7%) | 0 | 100% | 0 | 0 | 0 | 0 |
| Rungwe mpya | Rungwe mpya | 60 | 1(1.7%) | 100% | 0 | 0 | 0 | 0 | 0 |

**Key:** P=Prevalence, L=Low, M=Moderate, H=Heavy

Table 18: Prevalence and intensity of soil-transmitted helminths at Kasulu district council

| **Ward** | **School** | **N** | ***A. lumbricoides*** | | | | ***T. trichiura*** | | | | ***Hookworm*** | | | |
| --- | --- | --- | --- | --- | --- | --- | --- | --- | --- | --- | --- | --- | --- | --- |
|  |  |  | P | L | M | H | P | L | M | H | P | L | M | H |
| Buhoro | Kibilizi | 60 | 0 | 0 | 0 | 0 | 0 | 0 | 0 | 0 | 0 | 0 | 0 | 0 |
| Kasulu | Kigadye | 60 | 0 | 0 | 0 | 0 | 0 | 0 | 0 | 0 | 0 | 0 | 0 | 0 |
| Kasulu | Kitibitibi | 60 | 0 | 0 | 0 | 0 | 0 | 0 | 0 | 0 | 0 | 0 | 0 | 0 |
| Kasulu | Kumtundu | 60 | 0 | 0 | 0 | 0 | 0 | 0 | 0 | 0 | 0 | 0 | 0 | 0 |
| Rungwe mpya | Rungwe mpya | 60 | 0 | 0 | 0 | 0 | 1(1.7%) | 0 | 0 | 0 | 0 | 0 | 0 | 0 |

**Key:** P=Prevalence, L=Low, M=Moderate, H=Heavy

**Kibondo district council**

Table 19: Prevalence and intensities of schistosomiasis at Kibondo district council

| **Ward** | **School** | **N** | ***S. mansoni*** | | | | ***S. haematobium*** | | |
| --- | --- | --- | --- | --- | --- | --- | --- | --- | --- |
|  |  |  | P | L | M | H | P | Low | Heavy |
| Busagara | Kumshindwi | 60 | 3(5%) | 33.3% | 66.7% | 0 | 2(3.3%) | 0 | 100% |
| Busunzu | Nyalulanga | 60 | 17(28.3%) | 41.1% | 58.9 | 0 | 2(3.3%) | 100% | 0 |
| Kumsega | Kibuye | 60 | 7(11.7%) | 57.1% | 42.8% | 0 | 1(1.7%) | 100% | 0 |
| Mabamba | Nyakasado | 60 | 27(45%) | 29.6% | 29.6% | 40.7% | 0 | 0 | 0 |
| Rugongwe | Magarama | 60 | 28(46.7%) | 46.4% | 25% | 28.6% | 3(5%) | 100% | 0 |

**Key:** P=Prevalence, L=Low, M=Moderate, H=Heavy

Table 20: Prevalence and intensity of soil-transmitted helminths at Kibondo district council

| **Ward** | **School** | **N** | ***A. lumbricoides*** | | | | ***T. trichiura*** | | | | ***Hookworm*** | | | |
| --- | --- | --- | --- | --- | --- | --- | --- | --- | --- | --- | --- | --- | --- | --- |
|  |  |  | P | L | M | H | P | L | M | H | P | L | M | H |
| Busagara | Kumshindwi | 60 | 0 | 0 | 0 |  | 0 | 0 | 0 | 0 | 7(11.7%) | 100% | 0 | 0 |
| Busunzu | Nyalulanga | 60 | 0 | 0 | 0 |  | 0 | 0 | 0 | 0 | 0 | 0 | 0 | 0 |
| Kumsega | Kibuye | 60 | 0 | 0 | 0 |  | 0 | 0 | 0 | 0 | 3(5%) | 100% | 0 | 0 |
| Mabamba | Nyakasando | 60 | 1(1.7%) | 0 | 100% | 0 | 0 | 0 | 0 | 0 | 9(15%) | 100% | 0 | 0 |
| Rugongwe | Magarama | 60 | 1(1.7%) | 100% | 0 |  | 0 | 0 | 0 | 0 | 6(10%) | 100% | 0 | 0 |

**Key:** P=Prevalence, L=Low, M=Moderate, H=Heavy

**Kigoma district council**

Table 21: Prevalence and intensities of schistosomiasis at Kibondo district council

| **Ward** | **School** | **N** | ***S. mansoni*** | | | | ***S. haematobium*** | | |
| --- | --- | --- | --- | --- | --- | --- | --- | --- | --- |
|  |  |  | P | L | M | H | P | Low | Heavy |
| Kagunga | Zashe | 60 | 8(13.3%) | 25% | 75% | 0 | 0 | 0 | 0 |
| Mwamgongo | Legeza | 60 | 17(28.3%) | 70.6% | 23.5% | 5.9% | 0 | 0 | 0 |
| Simbo | Kaseke | 60 | 8(13.3%) | 50% | 37.5% | 12.5% | 0 | 0 | 0 |
| Ziwani | Mara | 60 | 2(3.3%) | 0 | 100% | 0 | 0 | 0 | 0 |
| Ziwani | Kalalangabo | 60 | 2(3.3%) | 100% | 0 | 0 | 0 | 0 | 0 |

**Key:** P=Prevalence, L=Low, M=Moderate, H=Heavy

Table 22: Prevalence and intensity of soil-transmitted helminths at Kibondo district council

| **Ward** | **School** | **N** | ***A. lumbricoides*** | | | | ***T. trichiura*** | | | | ***Hookworm*** | | | |
| --- | --- | --- | --- | --- | --- | --- | --- | --- | --- | --- | --- | --- | --- | --- |
|  |  |  | P | L | M | H | P | L | M | H | P | L | M | H |
| Kagunga | Zashe | 60 | 0 | 0 | 0 | 0 | 0 | 0 | 0 | 0 | 0 | 0 | 0 | 0 |
| Mwamgongo | Legeza | 60 | 18(30%) | 100% | 0 | 0 | 2(3.3%) | 100% | 0 | 0 | 0 | 0 | 0 | 0 |
| Simbo | Kaseke | 60 | 0 | 0 | 0 | 0 | 1(1.7%) | 100% | 0 | 0 | 0 | 0 | 0 | 0 |
| Ziwani | Mara | 60 | 0 | 0 | 0 | 0 | 0 | 0 | 0 | 0 | 0 | 0 | 0 | 0 |
| Ziwani | Kalalangabo | 60 | 0 | 0 | 0 | 0 | 0 | 0 | 0 | 0 | 0 | 0 | 0 | 0 |

**Key:** P=Prevalence, L=Low, M=Moderate, H=Heavy

**Kigoma municipal council**

Table 23: Prevalence and intensities of schistosomiasis at Kigoma municipal council

| **Ward** | **School** | **N** | ***S. mansoni*** | | | | ***S. haematobium*** | | |
| --- | --- | --- | --- | --- | --- | --- | --- | --- | --- |
|  |  |  | P | L | M | H | P | Low | Heavy |
| Bangwe | Bangwe | 60 | 5(8.3%) | 40% | 60% | 0 | 0 | 0 | 0 |
| Bangwe | Mkapa | 60 | 1(1.7%) | 100% | 0 | 0 | 0 | 0 | 0 |
| Kibilizi | Butunga | 60 | 4(6.5%) | 50% | 50% | 0 | 0 | 0 | 0 |
| Kibilizi | Kibilizi | 60 | 8(13.3%) | 50% | 50^ | 0 | 0 | 0 | 0 |
| Machinjioni | Kichangachui | 58 | 4(6.9%) | 25% | 75% | 0 | 0 | 0 | 0 |

**Key:** P=Prevalence, L=Low, M=Moderate, H=Heavy

Table 24: Prevalence and intensity of soil-transmitted helminths at Kigoma municipal council

| **Ward** | **School** | **N** | ***A. lumbricoides*** | | | | ***T. trichiura*** | | | | ***Hookworm*** | | | |
| --- | --- | --- | --- | --- | --- | --- | --- | --- | --- | --- | --- | --- | --- | --- |
|  |  |  | P | L | M | H | P | L | M | H | P | L | M | H |
| Bangwe | Bangwe | 60 | 0 | 0 | 0 | 0 | 0 | 0 | 0 | 0 | 0 | 0 | 0 | 0 |
| Bangwe | Mkapa | 60 | 0 | 0 | 0 | 0 | 0 | 0 | 0 | 0 | 0 | 0 | 0 | 0 |
| Kibilizi | Butunga | 60 | 0 | 0 | 0 | 0 | 0 | 0 | 0 | 0 | 0 | 0 | 0 | 0 |
| Kibilizi | Kibilizi | 60 | 0 | 0 | 0 | 0 | 0 | 0 | 0 | 0 | 0 | 0 | 0 | 0 |
| Machinjioni | Kichangachui | 58 | 0 | 0 | 0 | 0 | 0 | 0 | 0 | 0 | 3(5.2%) | 100% | 0 | 0 |

**Key:** P=Prevalence, L=Low, M=Moderate, H=Heavy


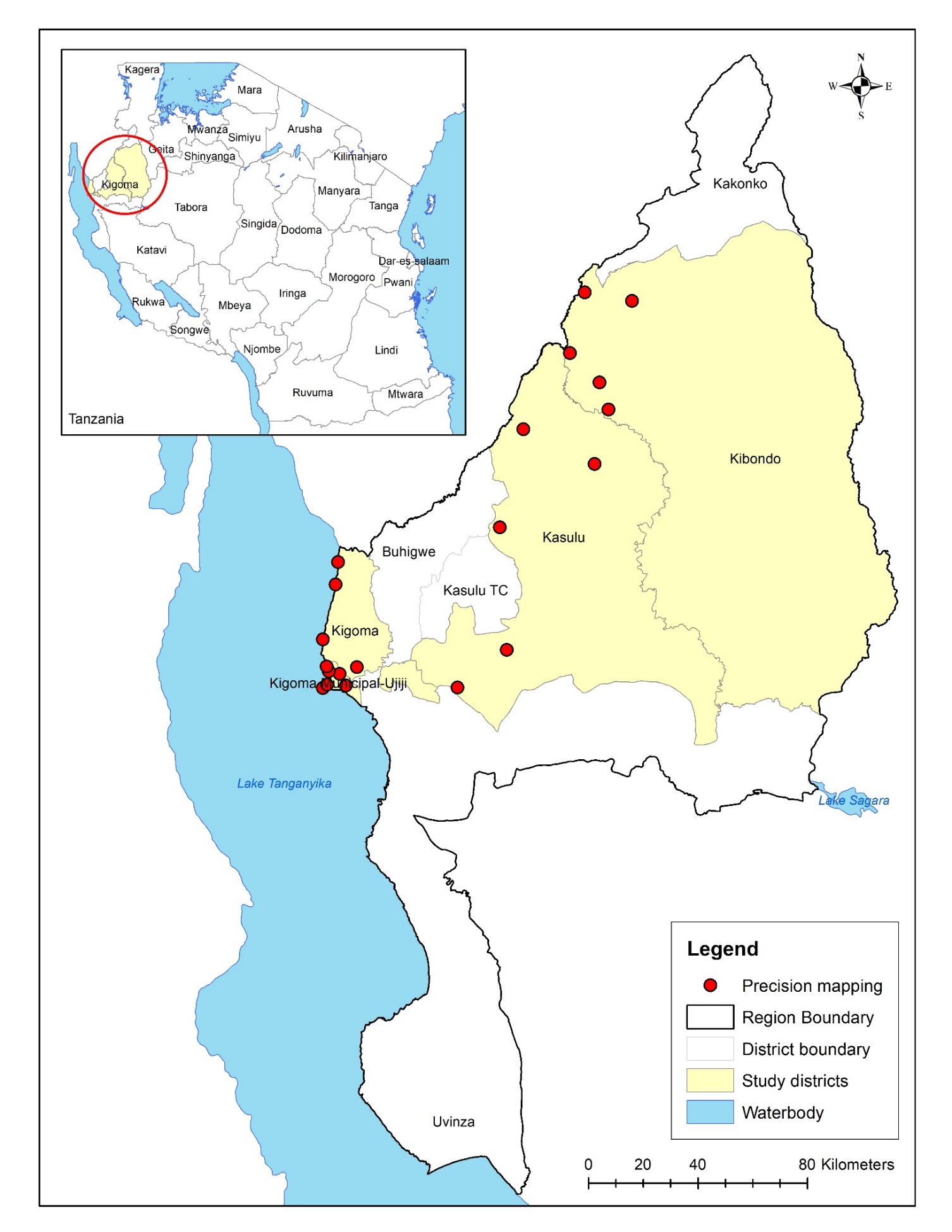


Figure 3: Geographical location of school involved in precision mapping in four districts of Kigoma, region, Western Tanzania

**Shinyanga region**

**Kahama district council**

Table 25: Prevalence and intensities of schistosomiasis at Kahama district council

| **Ward** | **School** | **N** | ***S. mansoni*** | | | | ***S. haematobium*** | | |
| --- | --- | --- | --- | --- | --- | --- | --- | --- | --- |
|  |  |  | P | L | M | H | P | Low | Heavy |
| Isagehe | Bukooba | 60 | 0 | 0 | 0 | 0 | 3 (5%) | 100% | 0 |
| Kagongwa | Gembe | 60 | 0 | 0 | 0 | 0 | 3(5%) | 100% | 0 |
| Kinaga | Nduku | 60 | 0 | 0 | 0 | 0 | 4(6.7%) | 100% | 0 |
| Yandekwa | Nyandekwa | 60 | 0 | 0 | 0 | 0 | 5(8.3%) | 100% | 0 |
| Zongomera | Guido | 60 | 0 | 0 | 0 | 0 | 5(8.3%) | 60% | 40% |

**Key:** P=Prevalence, L=Low, M=Moderate, H=Heavy

Table 26: Prevalence and intensity of soil-transmitted helminths at Kahama district council

| **Ward** | **School** | **N** | ***A. lumbricoides*** | | | | ***T. trichiura*** | | | | ***Hookworm*** | | | |
| --- | --- | --- | --- | --- | --- | --- | --- | --- | --- | --- | --- | --- | --- | --- |
|  |  |  | P | L | M | H | P | L | M | H | P | L | M | H |
| Isagehe | Bukooba | 60 | 0 | 0 | 0 | 0 | 0 | 0 | 0 | 0 | 0 | 0 | 0 | 0 |
| Kagongwa | Gembe | 60 | 0 | 0 | 0 | 0 | 0 | 0 | 0 | 0 | 8(13.3%) | 100% | 0 | 0 |
| Kinaga | Nduku | 60 | 0 | 0 | 0 | 0 | 0 | 0 | 0 | 0 | 4(6.7%) | 100% | 0 | 0 |
| Yandekwa | Nyandekwa | 60 | 0 | 0 | 0 | 0 | 0 | 0 | 0 | 0 | 4(6.7%) | 100% | 0 | 0 |
| Zongomera | Guido | 58 | 0 | 0 | 0 | 0 | 0 | 0 | 0 | 0 | 4(6.7%) | 100% | 0 | 0 |

**Key:** P=Prevalence, L=Low, M=Moderate, H=Heavy

**Kishapu district council**

Table 27: Prevalence and intensities of schistosomiasis at Kishapu district council

| **Ward** | **School** | **N** | ***S. mansoni*** | | | | ***S. haematobium*** | | |
| --- | --- | --- | --- | --- | --- | --- | --- | --- | --- |
|  |  |  | P | L | M | H | P | Low | Heavy |
| Kishapu | Kishapu | 60 | 0 | 0 | 0 | 0 | 3(5%) | 100% | 0 |
| Mwakipoya | Ilula | 60 | 1(1.7%) | 100% | 0 | 0 | 2(3.3%) | 100% | 0 |
| Songwa | Seseko | 60 | 0 | 0 | 0 | 0 | 8(13.3%) | 87.5% | 12.5% |
| Kishapu | Lubanga | 60 | 0 | 0 | 0 | 0 | 9(15%) | 100% | 0 |
| Talaga | Jijongo | 60 | 0 | 0 | 0 | 0 | 8(13.3%) | 87.5% | 12.5% |

**Key:** P=Prevalence, L=Low, M=Moderate, H=Heavy

Table 28: Prevalence and intensity of soil-transmitted helminths at Kishapu district council

| **Ward** | **School** | **N** | ***A. lumbricoides*** | | | | ***T. trichiura*** | | | | ***Hookworm*** | | | |
| --- | --- | --- | --- | --- | --- | --- | --- | --- | --- | --- | --- | --- | --- | --- |
|  |  |  | P | L | M | H | P | L | M | H | P | L | M | H |
| Kishapu | Kishapu | 60 | 0 | 0 | 0 | 0 | 0 | 0 | 0 | 0 | 0 | 0 | 0 | 0 |
| Mwakipoya | Ilula | 60 | 0 | 0 | 0 | 0 | 0 | 0 | 0 | 0 | 0 | 0 | 0 | 0 |
| Songwa | Seseko | 60 | 0 | 0 | 0 | 0 | 0 | 0 | 0 | 0 | 0 | 0 | 0 | 0 |
| Kishapu | Lubanga | 60 | 0 | 0 | 0 | 0 | 0 | 0 | 0 | 0 | 0 | 0 | 0 | 0 |
| Talaga | Jijongo | 60 | 0 | 0 | 0 | 0 | 0 | 0 | 0 | 0 | 0 | 0 | 0 | 0 |

**Key:** P=Prevalence, L=Low, M=Moderate, H=Heavy

**Msalala district council**

Table 29: Prevalence and intensities of schistosomiasis at Msalala district council

| **Ward** | **School** | **N** | ***S. mansoni*** | | | | ***S. haematobium*** | | |
| --- | --- | --- | --- | --- | --- | --- | --- | --- | --- |
|  |  |  | P | L | M | H | P | Low | Heavy |
| Chela | Chela | 60 | 0 | 0 | 0 | 0 | 0 | 0 | 0 |
| Isaka | Shishinulu | 60 | 0 | 0 | 0 | 0 | 0 | 0 | 0 |
| Mega | Nyaminje | 60 | 2(3.3%) | 100% | 0 | 0 | 0 | 0 | 0 |
| Mwanase | Mwamandi | 60 | 0 | 0 | 0 | 0 | 0 | 0 | 0 |
| Ntobo | Wichamike | 60 | 0 | 0 | 0 | 0 | 0 | 0 | 0 |

**Key:** P=Prevalence, L=Low, M=Moderate, H=Heavy

Table 30: Prevalence and intensity of soil-transmitted helminths at Msalala district council

| **Ward** | **School** | **N** | ***A. lumbricoides*** | | | | ***T. trichiura*** | | | | ***Hookworm*** | | | |
| --- | --- | --- | --- | --- | --- | --- | --- | --- | --- | --- | --- | --- | --- | --- |
|  |  |  | P | L | M | H | P | L | M | H | P | L | M | H |
| Chela | Chela | 60 | 0 | 0 | 0 | 0 | 0 | 0 | 0 | 0 | 2(3.3%) | 100% | 0 | 0 |
| Isaka | Shishinulu | 60 | 0 | 0 | 0 | 0 | 0 | 0 | 0 | 0 | 4(6.7%) | 100% | 0 | 0 |
| Mega | Nyaminje | 60 | 0 | 0 | 0 | 0 | 0 | 0 | 0 | 0 | 5(8.3%) | 100% | 0 | 0 |
| Mwanase | Mwamandi | 60 | 0 | 0 | 0 | 0 | 0 | 0 | 0 | 0 | 9(15%) | 100% | 0 | 0 |
| Ntobo | Wichamike | 58 | 0 | 0 | 0 | 0 | 0 | 0 | 0 | 0 | 0 | 0 | 0 | 0 |

**Key:** P=Prevalence, L=Low, M=Moderate, H=Heavy

**Ushetu district council**

Table 31: Prevalence and intensities of schistosomiasis Ushetu district council

| **Ward** | **School** | **N** | ***S. mansoni*** | | | | ***S. haematobium*** | | |
| --- | --- | --- | --- | --- | --- | --- | --- | --- | --- |
|  |  |  | P | L | M | H | P | Low | Heavy |
| Bulunga | Bukale | 60 | 0 | 0 | 0 | 0 | 8(13.3%) | 62.5% | 37.5% |
| Kisuke | Itumbo | 60 | 3(5%) | 33.3% | 66.7% | 0 | 4(6.7%) | 100% | 0 |
| Ulewe | Kalo | 60 | 0 | 0 | 0 | 0 | 3(5%) | 100% | 0 |
| Mapamba | Mapamba | 60 | 0 | 0 | 0 | 0 | 0 | 0 | 0 |
| Nyamilangano | Nyamilangano | 60 | 0 | 0 | 0 | 0 | 2(3.3/ | 50% | 50% |

**Key:** P=Prevalence, L=Low, M=Moderate, H=Heavy

Table 32: Prevalence and intensity of soil-transmitted helminths Ushetu district council

| **Ward** | **School** | **N** | ***A. lumbricoides*** | | | | ***T. trichiura*** | | | | ***Hookworm*** | | | |
| --- | --- | --- | --- | --- | --- | --- | --- | --- | --- | --- | --- | --- | --- | --- |
|  |  |  | P | L | M | H | P | L | M | H | P | L | M | H |
| Bulunga | Bukale | 60 | 0 | 0 | 0 | 0 | 0 | 0 | 0 | 0 | 18(30%) | 100% | 0 | 0 |
| Kisuke | Itumbo | 60 | 0 | 0 | 0 | 0 | 0 | 0 | 0 | 0 | 16(26.7%) | 100% | 0 | 0 |
| Ulewe | Kalo | 60 | 0 | 0 | 0 | 0 | 0 | 0 | 0 | 0 | 4(6.7%) | 100% | 0 | 0 |
| Mapamba | Mapamba | 60 | 0 | 0 | 0 | 0 | 0 | 0 | 0 | 0 | 2(3.3%) | 100% | 0 | 0 |
| Nyamilangano | Nyamilangano | 60 | 0 | 0 | 0 | 0 | 0 | 0 | 0 | 0 | 2(3.3%) | 100% | 0 | 0 |

**Key:** P=Prevalence, L=Low, M=Moderate, H=Heavy

**Shinyanga district council**

Table 33: Prevalence and intensities of schistosomiasis at Shinyanga district council

| **Ward** | **School** | **N** | ***S. mansoni*** | | | | ***S. haematobium*** | | |
| --- | --- | --- | --- | --- | --- | --- | --- | --- | --- |
|  |  |  | P | L | M | H | P | Low | Heavy |
| Samuye | Ishinabulandi | 60 | 0 | 0 | 0 | 0 | 20(33.3%) | 65% | 35% |
| Didia | Bugisi | 60 | 0 | 0 | 0 | 0 | 0 | 0 | 0 |
| Iselamagazi | Iselamagazi | 60 | 0 | 0 | 0 | 0 | 3(5%) | 100% | 0 |
| Puni | Buyabi | 60 | 0 | 0 | 0 | 0 | 2(33%) | 100% | 0 |
| Lyabusalu | Lyabusalu A | 60 | 0 | 0 | 0 | 0 | 5(8.3%) | 60% | 40% |

**Key:** P=Prevalence, L=Low, M=Moderate, H=Heavy

Table 34: Prevalence and intensity of soil-transmitted helminths at Shinyanga district council

| **Ward** | **School** | **N** | ***A. lumbricoides*** | | | | ***T. trichiura*** | | | | ***Hookworm*** | | | |
| --- | --- | --- | --- | --- | --- | --- | --- | --- | --- | --- | --- | --- | --- | --- |
|  |  |  | P | L | M | H | P | L | M | H | P | L | M | H |
| Samuye | Ishinabulandi | 60 | 0 | 0 | 0 | 0 | 0 | 0 | 0 | 0 | 4(6.7%) | 100% | 0 | 0 |
| Didia | Bugisi | 60 | 0 | 0 | 0 | 0 | 0 | 0 | 0 | 0 | 6(10%) | 100% | 0 | 0 |
| Iselamagazi | Iselamagazi | 60 | 0 | 0 | 0 | 0 | 0 | 0 | 0 | 0 | 2(3.3%) | 100% | 0 | 0 |
| Puni | Buyabi | 60 | 0 | 0 | 0 | 0 | 0 | 0 | 0 | 0 | 0 | 0 | 0 | 0 |
| Lyabusalu | Lyabusalu A | 60 | 0 | 0 | 0 | 0 | 0 | 0 | 0 | 0 | 1(1.7%) | 100% | 0 | 0 |

**Key:** P=Prevalence, L=Low, M=Moderate, H=Heavy


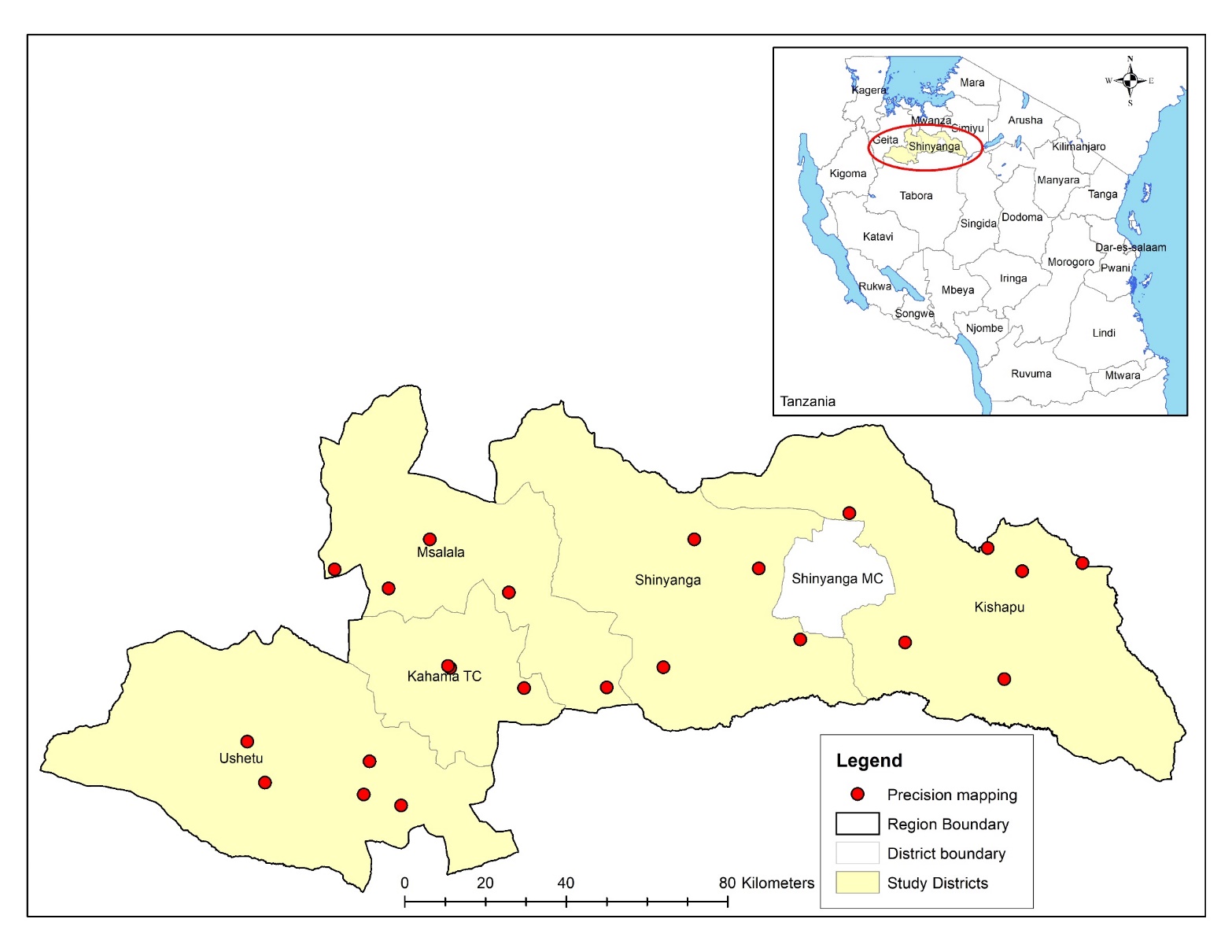


Figure 4: Geographical location of school involved in precision mapping in five districts of Shinyanga region, north-western Tanzania

**Mara region**

**Bunda district council**

Table 35: Prevalence and intensities of schistosomiasis at Bunda district council

| **Ward** | **School** | **N** | ***S. mansoni*** | | | | ***S. haematobium*** | | |
| --- | --- | --- | --- | --- | --- | --- | --- | --- | --- |
|  |  |  | P | L | M | H | P | Low | Heavy |
| Kisorya | Kisorya | 60 | 45(75%) | 33.3% | 35.6% | 31.1% | 0 | 0 | 0 |
| Butimba | Mwisemi | 60 | 5(8.3%) | 60% | 40% | 0 | 0 | 0 | 0 |
| Chitengule | Busambara | 60 | 16(26.7%) | 62.5% | 25% | 12.5% | 0 | 0 | 0 |
| Hunyari | Mariwanda | 60 | 0 | 0 | 0 | 0 | 0 | 0 | 0 |
| Mugeta | Nyang’aranga | 60 | 0 | 0 | 0 | 0 | 0 | 0 | 0 |

**Key:** P=Prevalence, L=Low, M=Moderate, H=Heavy

Table 36: Prevalence and intensity of soil-transmitted helminths at Bunda district council

| **Ward** | **School** | **N** | ***A. lumbricoides*** | | | | ***T. trichiura*** | | | | ***Hookworm*** | | | |
| --- | --- | --- | --- | --- | --- | --- | --- | --- | --- | --- | --- | --- | --- | --- |
|  |  |  | P | L | M | H | P | L | M | H | P | L | M | H |
| Kisorya | Kisorya | 60 | 0 | 0 | 0 | 0 | 0 | 0 | 0 | 0 | 43(71.7%) | 97.6% | 0 | 2.3% |
| Butimba | Mwisemi | 60 | 0 | 0 | 0 | 0 | 0 | 0 | 0 | 0 | 4(6.7%) | 100% | 0 | 0 |
| Chitengule | Busambara | 60 | 0 | 0 | 0 | 0 | 0 | 0 | 0 | 0 | 0 | 0 | 0 | 0 |
| Hunyari | Mariwanda | 60 | 0 | 0 | 0 | 0 | 0 | 0 | 0 | 0 | 0 | 0 | 0 | 0 |
| Mugeta | Nyang’aranga | 60 | 0 | 0 | 0 | 0 | 0 | 0 | 0 | 0 | 0 | 0 | 0 | 0 |

**Key:** P=Prevalence, L=Low, M=Moderate, H=Heavy

**Musoma district council**

Table 37: Prevalence and intensities of schistosomiasis at Musoma district council

| **Ward** | **School** | **N** | ***S. mansoni*** | | | | ***S. haematobium*** | | |
| --- | --- | --- | --- | --- | --- | --- | --- | --- | --- |
|  |  |  | P | L | M | H | P | Low | Heavy |
| Suguti | Suguti B | 60 | 35 (58.3% | 34.3% | 45.7% | 20% | 0 | 0 | 0 |
| Makojo | Chitare B | 60 | 12(20%) | 41.7% | 58.3% | 0 | 0 | 0 | 0 |
| Bukumi | Bukumi | 60 | 10(16.7%) | 10% | 70% | 20% | 0 | 0 | 0 |
| Musanja | Musanja | 60 | 4(6.7%) | 75% | 25% | 0 | 0 | 0 | 0 |
| Bugwema | Masinono | 60 | 1(1.7%) | 100% | 0 | 0 | 0 | 0 | 0 |

**Key:** P=Prevalence, L=Low, M=Moderate, H=Heavy

Table 38: Prevalence and intensity of soil-transmitted helminths at Musoma district council

| **Ward** | **School** | **N** | ***A. lumbricoides*** | | | | ***T. trichiura*** | | | | ***Hookworm*** | | | |
| --- | --- | --- | --- | --- | --- | --- | --- | --- | --- | --- | --- | --- | --- | --- |
|  |  |  | P | L | M | H | P | L | M | H | P | L | M | H |
| Suguti | Suguti B | 60 | 0 | 0 | 0 | 0 | 0 | 0 | 0 | 0 | 0 | 0 | 0 | 0 |
| Makojo | Chitare B | 60 | 0 | 0 | 0 | 0 | 0 | 0 | 0 | 0 | 2(3.3%) | 100% | 0 | 0 |
| Bukumi | Bukumi | 60 | 0 | 0 | 0 | 0 | 0 | 0 | 0 | 0 | 8(13.3%) | 75% | 25% | 0 |
| Musanja | Musanja | 60 | 0 | 0 | 0 | 0 | 1(1.7%) | 100% | 0 | 0 | 0 | 100% | 0 | 0 |
| Nyamilangano | Nyamilangano | 60 | 0 | 0 | 0 | 0 | 0 | 0 | 0 | 0 | 1(1.7%) | 100% | 0 | 0 |

**Key:** P=Prevalence, L=Low, M=Moderate, H=Heavy

**Musoma municipal council**

Table 39: Prevalence and intensities of schistosomiasis at Musoma municipal council

| **Ward** | **School** | **N** | ***S. mansoni*** | | | | ***S. haematobium*** | | |
| --- | --- | --- | --- | --- | --- | --- | --- | --- | --- |
|  |  |  | P | L | M | H | P | Low | Heavy |
| Kamunyonge | Kamunyonge | 60 | 7(11.7%) | 42.8% | 42.8% | 14.3% | 0 | 0 | 0 |
| Kwanga | Kwanga | 60 | 4(6.7%) | 25% | 25% | 50% | 0 | 0 | 0 |
| Mshikamano | Mshikamano | 60 | 2(3.3%) | 50% | 50% | 0 | 0 | 0 | 0 |
| Mwisenge | Mwisenge | 60 | 22(36.7%) | 27.3% | 45.5% | 27.3% | 0 | 0 | 0 |
| Nyasho | Nyasho | 60 | 5(8.3%) | 0 | 80% | 20% | 0 | 0 | 0 |

**Key:** P=Prevalence, L=Low, M=Moderate, H=Heavy

Table 40: Prevalence and intensity of soil-transmitted helminths at Musoma municipal council

| **Ward** | **School** | **N** | ***A. lumbricoides*** | | | | ***T. trichiura*** | | | | ***Hookworm*** | | | |
| --- | --- | --- | --- | --- | --- | --- | --- | --- | --- | --- | --- | --- | --- | --- |
|  |  |  | P | L | M | H | P | L | M | H | P | L | M | H |
| Kamunyonge | Kamunyonge | 60 | 1(1.7%) | 0 | 0 | 0 | 0 | 0 | 0 | 0 | 0 | 0 | 0 | 0 |
| Kwanga | Kwanga | 60 | 0 | 0 | 0 | 0 | 0 | 0 | 0 | 0 | 0 | 0 | 0 | 0 |
| Mshikamano | Mshikamano | 60 | 0 | 0 | 0 | 0 | 0 | 0 | 0 | 0 | 0 | 0 | 0 | 0 |
| Mwisenge | Mwisenge | 60 | 0 | 0 | 0 | 0 | 0 | 0 | 0 | 0 | 1(1.7%) | 100% | 0 | 0 |
| Nyasho | Nyasho | 60 | 0 | 0 | 0 | 0 | 0 | 0 | 0 | 0 | 0 | 0 | 0 | 0 |

**Key:** P=Prevalence, L=Low, M=Moderate, H=Heavy

**Tarime District council**

Table 41: Prevalence and intensities of schistosomiasis at Tarime district council

| **Ward** | **School** | **N** | ***S. mansoni*** | | | | ***S. haematobium*** | | |
| --- | --- | --- | --- | --- | --- | --- | --- | --- | --- |
|  |  |  | P | L | M | H | P | Low | Heavy |
| Bumera | Taisi | 60 | 0 | 0 | 0 | 0 | 2(3.3%) | 100% | 0 |
| Matongo | Kenyangi | 60 | 0 | 0 | 0 | 0 | 0 | 0 | 0 |
| Muriba | Bungurere | 60 | 0 | 0 | 0 | 0 | 1(1.7%) | 0 | 0 |
| Kwihancha | Karakatonga | 60 | 0 | 0 | 0 | 0 | 2(3.3%) | 100% | 0 |
| Binagi | Nyasaricho | 60 | 0 | 0 | 0 | 0 | 0 | 0 | 0 |

**Key:** P=Prevalence, L=Low, M=Moderate, H=Heavy

Table 42: Prevalence and intensity of soil-transmitted helminths at Tarime district council

| **Ward** | **School** | **N** | ***A. lumbricoides*** | | | | ***T. trichiura*** | | | | ***Hookworm*** | | | |
| --- | --- | --- | --- | --- | --- | --- | --- | --- | --- | --- | --- | --- | --- | --- |
|  |  |  | P | L | M | H | P | L | M | H | P | L | M | H |
| Bumera | Taisi | 60 | 0 | 0 | 0 | 0 | 0 | 0 | 0 | 0 | 22(36.7%) | 100% | 0 | 0 |
| Matongo | Kenyangi | 60 | 0 | 0 | 0 | 0 | 0 | 0 | 0 | 0 | 1(1.7%) | 100% | 0 | 0 |
| Muriba | Bungurere | 60 | 0 | 0 | 0 | 0 | 0 | 0 | 0 | 0 | 2(3.3%) | 100% | 0 | 0 |
| Kwihancha | Karakatonga | 60 | 0 | 0 | 0 | 0 | 0 | 0 | 0 | 0 | 3(5%) | 100% | 0 | 0 |
| Binagi | Nyasaricho | 60 | 0 | 0 | 0 | 0 | 0 | 0 | 0 | 0 | 1(1.7%) | 100% | 0 | 0 |

**Rorya district council**

Table 43: Prevalence and intensities of schistosomiasis at Rorya district council

| **Ward** | **School** | **N** | ***S. mansoni*** | | | | ***S. haematobium*** | | |
| --- | --- | --- | --- | --- | --- | --- | --- | --- | --- |
|  |  |  | P | L | M | H | P | Low | Heavy |
| Kisumwa | Kukona | 60 | 2(3.3%) | 50% | 50% | 0 | 8(13.3%) | 62.5% | 37.5% |
| Komuge | Kyamwene | 60 | 5(8.3%) | 40% | 60% | 0 | 4(6.7%) | 100% | 0 |
| Kyangasaga | Nyamugere | 60 | 36(60%) | 25% | 38.8% | 36.1% | 3(5%) | 100% | 0 |
| Nyamagaro | Bugendi B | 60 | 36(60%) | 27.8% | 22.2% | 50% | 0 | 0 | 0 |
| Kigunga | Luanda Kusemi | 60 | 6(10%) | 50% | 33% | 16.7% | 2(3.3/ | 50% | 50% |

**Key:** P=Prevalence, L=Low, M=Moderate, H=Heavy

Table 44: Prevalence and intensity of soil-transmitted helminths at Rorya district council

| **Ward** | **School** | **N** | ***A. lumbricoides*** | | | | ***T. trichiura*** | | | | ***Hookworm*** | | | |
| --- | --- | --- | --- | --- | --- | --- | --- | --- | --- | --- | --- | --- | --- | --- |
|  |  |  | P | L | M | H | P | L | M | H | P | L | M | H |
| Kisumwa | Kukona | 60 | 0 | 0 | 0 | 0 | 0 | 0 | 0 | 0 | 0 | 0 | 0 | 0 |
| Komuge | Kyamwene | 60 | 0 | 0 | 0 | 0 | 0 | 0 | 0 | 0 | 0 | 0 | 0 | 0 |
| Kyangasaga | Nyamugere | 60 | 0 | 0 | 0 | 0 | 0 | 0 | 0 | 0 | 0 | 0 | 0 | 0 |
| Nyamagaro | Bugendi B | 60 | 3(5%) | 66.7% | 33.3% | 0 | 0 | 0 | 0 | 0 | 0 | 0 | 0 | 0 |
| Kigunga | Luanda Kusemi | 60 | 0 | 0 | 0 | 0 | 0 | 0 | 0 | 0 | 0 | 0 | 0 | 0 |

**Key:** P=Prevalence, L=Low, M=Moderate, H=Heavy

**Serengeti district council**

Table 45: Prevalence and intensities of schistosomiasis at Serengeti district council

| **Ward** | **School** | **N** | ***S. mansoni*** | | | | ***S. haematobium*** | | |
| --- | --- | --- | --- | --- | --- | --- | --- | --- | --- |
|  |  |  | P | L | M | H | P | Low | Heavy |
| Natta | Nattabigo | 60 | 1(1.7%) | 100% | 0 | 0 | 1(1.7%) | 100% | 0 |
| Standi kuu | Mugumu | 60 | 2(3.3%) | 50% | 0 | 50% | 4(6.7%) | 50% | 50% |
| Kisaka | Borenga | 60 | 0 | 0 | 0 | 0 | 0 | 0 | 0 |
| Issenye | Nyamisingisi | 60 | 0 | 0 | 0 | 0 | 0 | 0 | 0 |
| Nyamatare | Rwingwani | 60 | 0 | 0 | 0 | 0 | 3(5%) | 66.6% | 33.3% |

**Key:** P=Prevalence, L=Low, M=Moderate, H=Heavy

Table 46: Prevalence and intensity of soil-transmitted helminths at Serengeti district council

| **Ward** | **School** | **N** | ***A. lumbricoides*** | | | | ***T. trichiura*** | | | | ***Hookworm*** | | | |
| --- | --- | --- | --- | --- | --- | --- | --- | --- | --- | --- | --- | --- | --- | --- |
|  |  |  | P | L | M | H | P | L | M | H | P | L | M | H |
| Natta | Nattabigo | 60 | 1(1.7%) | 100% | 0 | 0 | 0 | 0 | 0 | 0 | 4(6.7%) | 100% | 0 | 0 |
| Standi kuu | Mugumu | 60 | 0 | 0 | 0 | 0 | 0 | 0 | 0 | 0 | 6(10%) | 100% | 0 | 0 |
| Kisaka | Borenga | 60 | 1(1.7%) | 100% | 0 | 0 | 0 | 0 | 0 | 0 | 9(15%) | 100% | 0 | 0 |
| Issenye | Nyamisingisi | 60 | 0 | 0 | 0 | 0 | 0 | 0 | 0 | 0 | 1(1.7%) | 100% | 0 | 0 |
| Nyamatare | Rwingani | 60 | 0 | 0 | 0 | 0 | 0 | 0 | 0 | 0 | 4(6.7%) | 100% | 0 | 0 |

**Key:** P=Prevalence, L=Low, M=Moderate, H=Heavy


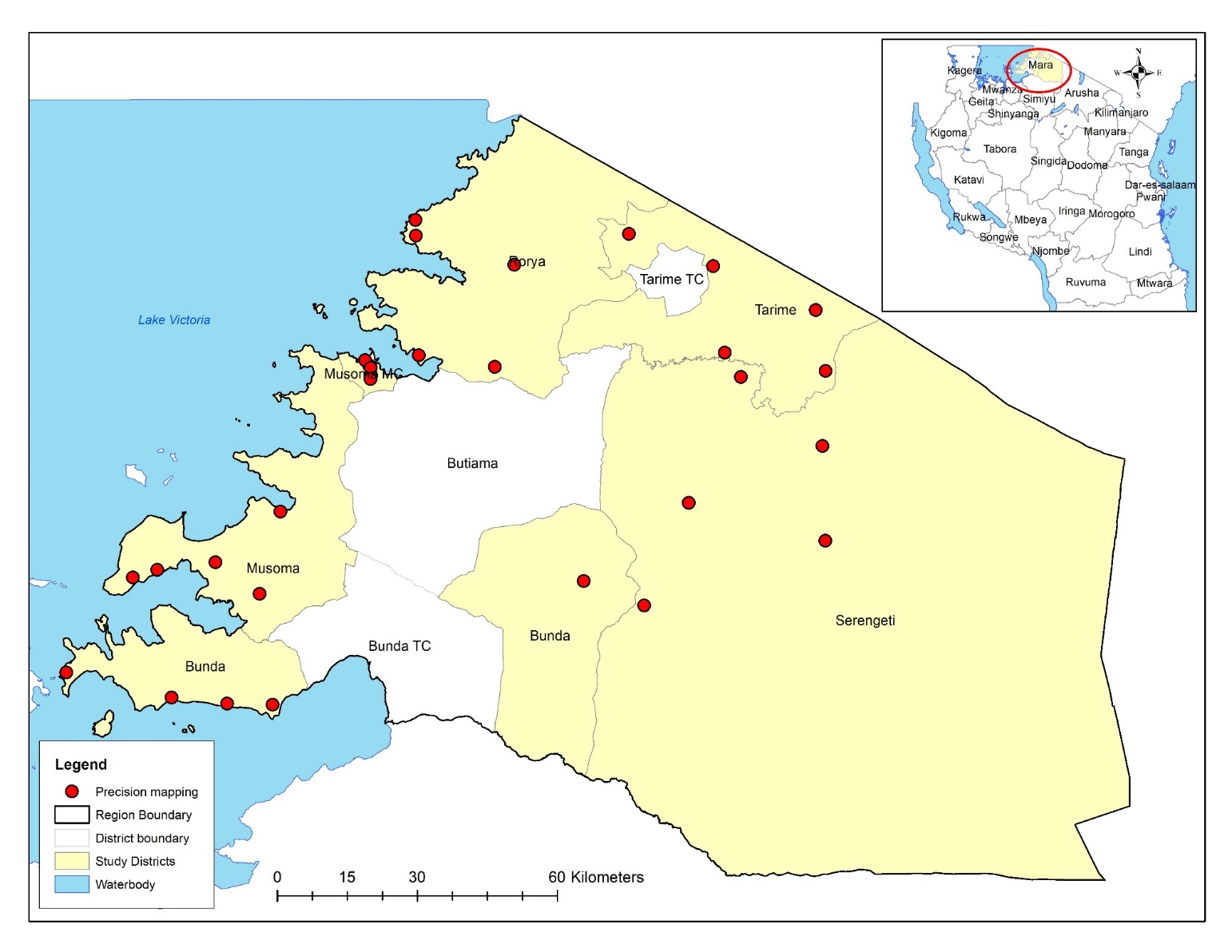


Figure 5: Geographical location of school involved in precision mapping in five districts of Mara region, north-western Tanzania

**Kagera region**

**Biharamulo district council**

Table 47: Prevalence and intensities of schistosomiasis at Biharamulo district council

| **Ward** | **School** | **N** | ***S. mansoni*** | | | | ***S. haematobium*** | | |
| --- | --- | --- | --- | --- | --- | --- | --- | --- | --- |
|  |  |  | P | L | M | H | P | Low | Heavy |
| Kabindi | Kabindi | 60 | 0 | 0 | 0 | 0 | 0 | 0 | 0 |
| Nyantakara | Iyengamulilo | 60 | 0 | 0 | 0 | 0 | 3(5%) | 100% | 0 |
| Nemba | Nemba | 60 | 0 | 0 | 0 | 0 | 8(13.3%) | 75% | 25% |
| Kaniha | Msalabani | 60 | 0 | 0 | 0 | 0 | 3(5%) | 100% | 0 |
| Nyantakara | Kasilo | 60 | 0 | 0 | 0 | 0 | 3(100%) | 0 | 0 |

**Key:** P=Prevalence, L=Low, M=Moderate, H=Heavy

Table 48: Prevalence and intensity of soil-transmitted helminths at Biharamulo district council

| **Ward** | **School** | **N** | ***A. lumbricoides*** | | | | ***T. trichiura*** | | | | ***Hookworm*** | | | |
| --- | --- | --- | --- | --- | --- | --- | --- | --- | --- | --- | --- | --- | --- | --- |
|  |  |  | P | L | M | H | P | L | M | H | P | L | M | H |
| Kabindi | Kabindi | 60 | 0 | 0 | 0 | 0 | 0 | 0 | 0 | 0 | 14(23.3%) | 100% | 0 | 0 |
| Nyantakara | Iyengamulilo | 60 | 0 | 0 | 0 | 0 | 0 | 0 | 0 | 0 | 29(48.3%) | 89.6% | 10.3% | 0 |
| Nemba | Nemba | 60 | 0 | 0 | 0 | 0 | 0 | 0 | 0 | 0 | 26(43.3%) | 100% | 0 | 0 |
| Kaniha | Msalabani | 60 | 0 | 0 | 0 | 0 | 1(1.7%) | 100% | 0 | 0 | 28(46.7%) | 100% | 0 | 0 |
| Nyantakara | Kasilo | 60 | 0 | 0 | 0 | 0 | 0 | 0 | 0 | 0 | 20(33.3%) | 100% | 0 | 0 |

**Key:** P=Prevalence, L=Low, M=Moderate, H=Heavy

**Bukoba district council**

Table 49: Prevalence and intensities of schistosomiasis at Bukoba district council

| **Ward** | **School** | **N** | ***S. mansoni*** | | | | ***S. haematobium*** | | |
| --- | --- | --- | --- | --- | --- | --- | --- | --- | --- |
|  |  |  | P | L | M | H | P | Low | Heavy |
| Rubafu | Rwima | 60 | 5(8.3%) | 40% | 40% | 20% | 0 | 0 | 0 |
| Kishanje | Iluhya | 60 | 0 | 0 | 0 | 0 | 0 | 0 | 0 |
| Izimbya | Kaleego | 60 | 0 | 0 | 0 | 0 | 0 | 0 | 0 |
| Nyakato | Ibosa | 60 | 0 | 0 | 0 | 0 | 0 | 0 | 0 |
| Rukoma | Karama | 60 | 3(5%) | 33.3% | 66.6% | 0 | 0 | 0 | 0 |

**Key:** P=Prevalence, L=Low, M=Moderate, H=Heavy

Table 50: Prevalence and intensity of soil-transmitted helminths at Bukoba district council

| **Ward** | **School** | **N** | ***A. lumbricoides*** | | | | ***T. trichiura*** | | | | ***Hookworm*** | | | |
| --- | --- | --- | --- | --- | --- | --- | --- | --- | --- | --- | --- | --- | --- | --- |
|  |  |  | P | L | M | H | P | L | M | H | P | L | M | H |
| Rubafu | Rwima | 60 | 36(60%) | 94.4% | 5.6% | 0 | 20(33.3%) | 0 | 0 | 0 | 1(1.7%) | 100% | 0 | 0 |
| Kishanje | Iluhya | 60 | 23(38.3%) | 86.9% | 13% | 0 | 18(30%) | 100% | 0 | 0 | 2(3.3%) | 100% | 0 | 0 |
| Izimbya | Kaleego | 60 | 0 | 0 | 0 | 0 | 0 | 0 | 0 | 0 | 0 | 0 | 0 | 0 |
| Nyakato | Ibosa | 60 | 32(53.3%) | 90.6% | 9.4% | 0 | 11(18.3%) | 100% | 0 | 0 | 1(1.7%) | 0 | 0 | 0 |
| Rukoma | Karama | 60 | 0 | 0 | 0 | 0 | 0 | 0 | 0 | 0 | 5(100%) | 0 | 0 | 0 |

**Key:** P=Prevalence, L=Low, M=Moderate, H=Heavy

**Karagwe district council**

Table 51: Prevalence and intensities of schistosomiasis at Karagwe district council

| **Ward** | **School** | **N** | ***S. mansoni*** | | | | ***S. haematobium*** | | |
| --- | --- | --- | --- | --- | --- | --- | --- | --- | --- |
|  |  |  | P | L | M | H | P | Low | Heavy |
| Kamagambo | Kiregete | 60 | 1(1.7%) | 100% | 0 | 0 | 0 | 0 | 0 |
| Bugene | Bujuruga | 60 | 0 | 0 | 0 | 0 | 0 | 0 | 0 |
| Nyakahanga | Mato | 60 | 0 | 0 | 0 | 0 | 0 | 0 | 0 |
| Rugu | Ruhita | 60 | 0 | 0 | 0 | 0 | 0 | 0 | 0 |
| Rugu | Kahanga | 60 | 3(5%) | 33.3% | 66.6% | 0 | 0 | 0 | 0 |

**Key:** P=Prevalence, L=Low, M=Moderate, H=Heavy

Table 52: Prevalence and intensity of soil-transmitted helminths at Karagwe district council

| **Ward** | **School** | **N** | ***A. lumbricoides*** | | | | ***T. trichiura*** | | | | ***Hookworm*** | | | |
| --- | --- | --- | --- | --- | --- | --- | --- | --- | --- | --- | --- | --- | --- | --- |
|  |  |  | P | L | M | H | P | L | M | H | P | L | M | H |
| Kamagambo | Kiregete | 60 | 1(1.7%) | 0 | 100% | 0 | 0 | 0 | 0 | 0 | 0 | 0 | 0 | 0 |
| Bugene | Bujuruga | 60 | 0 | 0 | 0 | 0 | 0 | 0 | 0 | 0 | 0 | 0 | 0 | 0 |
| Nyakahanga | Mato | 60 | 0 | 0 | 0 | 0 | 0 | 0 | 0 | 0 | 0 | 0 | 0 | 0 |
| Rugu | Ruhita | 60 | 0 | 0 | 0 | 0 | 0 | 0 | 0 | 0 | 0 | 0 | 0 | 0 |
| Rugu | Kahanga | 60 | 0 | 0 | 0 | 0 | 0 | 0 | 0 | 0 | 0 | 0 | 0 | 0 |

**Key:** P=Prevalence, L=Low, M=Moderate, H=Heavy

**Misenyi district council**

Table 53: Prevalence and intensities of schistosomiasis at Misenyi district council

| **Ward** | **School** | **N** | ***S. mansoni*** | | | | ***S. haematobium*** | | |
| --- | --- | --- | --- | --- | --- | --- | --- | --- | --- |
|  |  |  | P | L | M | H | P | Low | Heavy |
| Ishunju | Ishunju | 60 | 0 | 0 | 0 | 0 | 9(15%) | 77.8% | 22.2% |
| Kasambya | Bunazi | 60 | 0 | 0 | 0 | 0 | 0 | 0 | 0 |
| Mushasha | Bulambo | 60 | 0 | 0 | 0 | 0 | 6(10%) | 50% | 50% |
| Kashenye | Kashenye | 60 | 0 | 0 | 0 | 0 | 17(28.3%) | 100% | 0 |
| Byeju | Byeju | 60 | 0 | 0 | 0 | 0 | 0 | 0 | 0 |

**Key:** P=Prevalence, L=Low, M=Moderate, H=Heavy

Table 54: Prevalence and intensity of soil-transmitted helminths at Misenyi district council

| **Ward** | **School** | **N** | ***A. lumbricoides*** | | | | ***T. trichiura*** | | | | ***Hookworm*** | | | |
| --- | --- | --- | --- | --- | --- | --- | --- | --- | --- | --- | --- | --- | --- | --- |
|  |  |  | P | L | M | H | P | L | M | H | P | L | M | H |
| Ishunju | Ishunju | 60 | 12(20%) | 100% | 0 | 0 | 22(36.7%) | 100% | 0 | 0 | 10(16.7%) | 100% | 0 | 0 |
| Kasambya | Bunazi | 60 | 0 | 0 | 0 | 0 | 0 | 0 | 0 | 0 | 0 | 0 | 0 | 0 |
| Mushasha | Bulembo | 60 | 39(65%) | 100% | 0 | 0 | 3(5%) | 100% | 0 | 0 | 46(76.7%) | 97.8% | 2.2% | 0 |
| Kashenye | Kashenye | 60 | 2(3.3%) | 100% | 0 | 0 | 15(25%) | 100% | 0 | 0 | 6(10%) | 100% | 0 | 0 |
| Byeju | Byeju | 60 | 1(1.7%) | 100% | 0 | 0 | 1(1.7%) | 100% | 0 | 0 | 5(8.3%) | 100% | 0 | 0 |

**Key:** P=Prevalence, L=Low, M=Moderate, H=Heavy

**Muleba district council**

Table 55: Prevalence and intensities of schistosomiasis at Muleba district council

| **Ward** | **School** | **N** | ***S. mansoni*** | | | | ***S. haematobium*** | | |
| --- | --- | --- | --- | --- | --- | --- | --- | --- | --- |
|  |  |  | P | L | M | H | P | Low | Heavy |
| Ikuza | Rwazi | 60 | 29(48.3%) | 24.1% | 37.9% | 37.9% | 0 | 0 | 0 |
| Bunyozi | Bunyozi | 60 | 32(53.3%) | 31.3% | 50% | 18.8% | 0 | 0 | 0 |
| Izigo | Rwahahoza | 60 | 0 | 0 | 0 | 0 | 0 | 0 | 0 |
| Katoke | Kimbugu | 60 | 5(8.3%) | 80% | 20% | 0 | 1(1.7%) | 100% | 0 |
| Ijumbi | Ijumbi | 60 | 0 | 0 | 0 | 0 | 0 | 0 | 0 |

**Key:** P=Prevalence, L=Low, M=Moderate, H=Heavy

Table 56: Prevalence and intensity of soil-transmitted helminths at Muleba district council

| **Ward** | **School** | **N** | ***A. lumbricoides*** | | | | ***T. trichiura*** | | | | ***Hookworm*** | | | |
| --- | --- | --- | --- | --- | --- | --- | --- | --- | --- | --- | --- | --- | --- | --- |
|  |  |  | P | L | M | H | P | L | M | H | P | L | M | H |
| Ikuza | Rwazi | 60 | 19(31.7%) | 89.5% | 10.5% | 0 | 0 | 0 | 0 | 0 | 0 | 0 | 0 | 0 |
| Bunyozi | Bunyozi | 60 | 1(1.7%) | 100% | 0 | 0 | 0 | 0 | 0 | 0 | 0 | 0 | 0 | 0 |
| Izigo | Rwahahoza | 60 | 10(16.7%) | 100% | 0 | 0 | 2(3.3%) | 100% | 0 | 0 | 0 | 0 | 0 | 0 |
| Katoke | Kimbugu | 60 | 18(30%) | 77.8% | 22.2% | 0 | 12(20%) | 100% | 0 | 0 | 0 | 0 | 0 | 0 |
| Ijumbi | Ijumbi | 60 | 7(11.7%) | 85.7% | 14.3% | 0 | 0 | 0 | 0 | 0 | 0 | 0 | 0 | 0 |

**Key:** P=Prevalence, L=Low, M=Moderate, H=Heavy

**Ngara district**

Table 57: Prevalence and intensities of schistosomiasis at Ngara district council

| **Ward** | **School** | **N** | ***S. mansoni*** | | | | ***S. haematobium*** | | |
| --- | --- | --- | --- | --- | --- | --- | --- | --- | --- |
|  |  |  | P | L | M | H | P | Low | Heavy |
| Kabanga | Ngundusi | 60 | 0 | 0 | 0 | 0 | 0 | 0 | 0 |
| Buririro | Ntobeye | 60 | 0 | 0 | 0 | 0 | 0 | 0 | 0 |
| Murukulazo | Busumo | 60 | 1(1.7%) | 100% | 0 | 0 | 0 | 0 | 0 |
| Buririro | Kigarama | 60 | 0 | 0 | 0 | 0 | 0 | 0 | 0 |
| Rulenge | Nyakahanga | 60 | 0 | 0 | 0 | 0 | 0 | 0 | 0 |

**Key:** P=Prevalence, L=Low, M=Moderate, H=Heavy

Table 58: Prevalence and intensity of soil-transmitted helminths at Ngara district council

| **Ward** | **School** | **N** | ***A. lumbricoides*** | | | | ***T. trichiura*** | | | | ***Hookworm*** | | | |
| --- | --- | --- | --- | --- | --- | --- | --- | --- | --- | --- | --- | --- | --- | --- |
|  |  |  | P | L | M | H | P | L | M | H | P | L | M | H |
| Kabanga | Ngundusi | 60 | 11(18.3%) | 100% |  |  | 4(6.7%) | 100% | 0 | 0 | 0 | 0 | 0 | 0 |
| Buriririo | Ntobeye | 60 | 0 | 0 | 0 | 0 | 0 | 0 | 0 | 0 | 0 | 0 | 0 | 0 |
| Murukulazo | Busumo | 60 | 0 | 0 | 0 | 0 | 0 | 0 | 0 | 0 | 0 | 0 | 0 | 0 |
| Buririro | Kigarama | 60 | 0 | 0 | 0 | 0 | 0 | 0 | 0 | 0 | 0 | 0 | 0 | 0 |
| Rulenge | Nyakahanga | 60 | 0 | 0 | 0 | 0 | 0 | 0 | 0 | 0 | 0 | 0 | 0 | 0 |

**Key:** P=Prevalence, L=Low, M=Moderate, H=Heavy


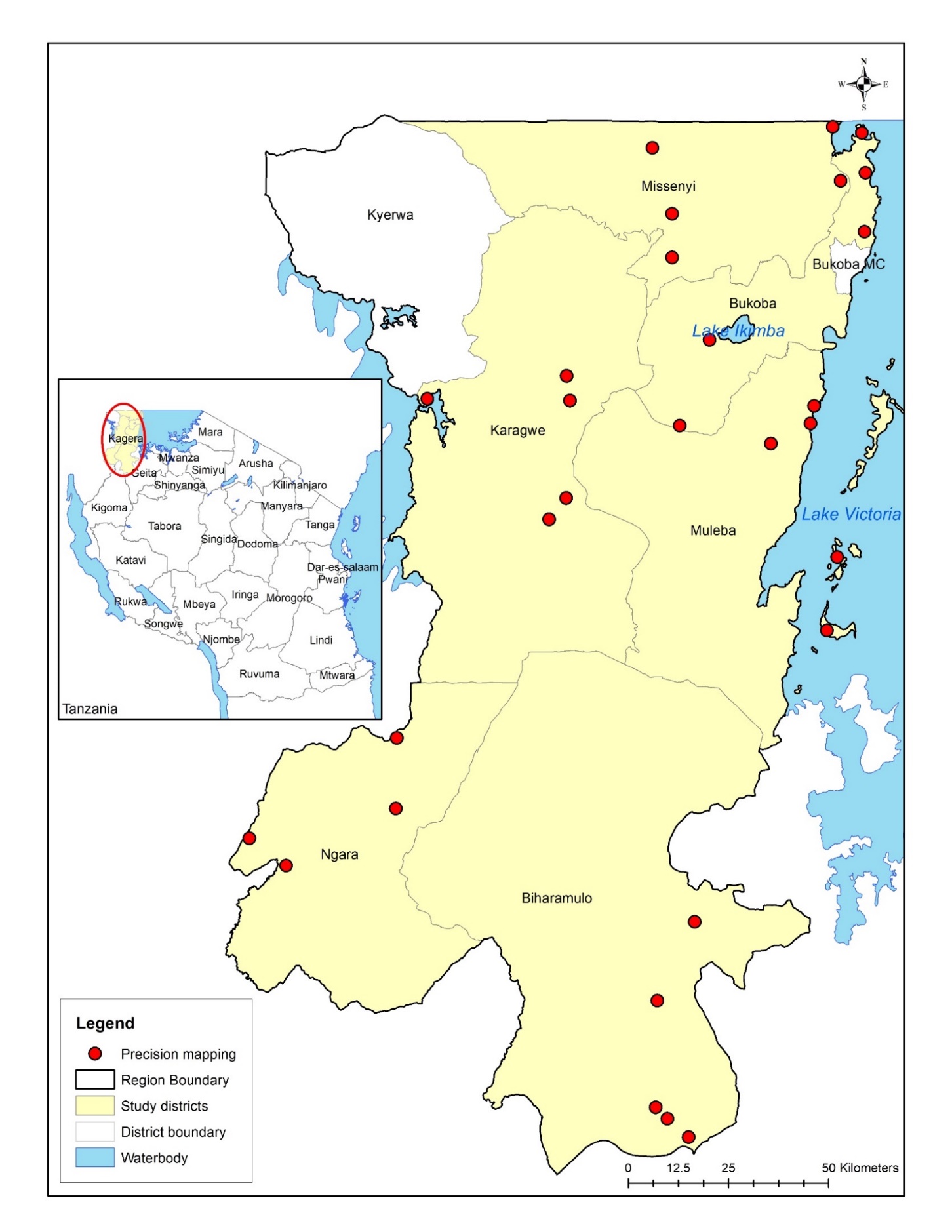


Figure 6: Geographical location of school involved in precision mapping in five districts of Kagera region, north-western Tanzania
